# Environmental regulation, industrial structure and energy efficiency: Evidence from 30 provinces in China

**DOI:** 10.1371/journal.pone.0299731

**Published:** 2024-05-20

**Authors:** Haicheng Zhu, Penghui Cai, Hao Fang, Zhengyu Zhu, Yao Li, Ruoqing Zhu

**Affiliations:** 1 School of Economics, Zhejiang University of Finance & Economics, Hangzhou, China; 2 Department of Sinology, KU Leuven, Leuven, Belgium; 3 Architecture and Design Department, University of Genoa, Genoa, Italy; Qufu Normal University, CHINA

## Abstract

The government’s environmental protection policy can significantly contribute to alleviating resource shortages and curbing environmental pollution, but the impact of various policy instruments implemented by the government on energy efficiency is unclear. Based on the panel data of 30 provinces in China from 2005 to 2021, this paper analyses the impact of environmental regulation and the industrial structure on energy efficiency from the perspective of resource taxes. The U-shaped relationship between environmental regulation and energy efficiency and between the optimization of industrial structure can significantly improve energy efficiency, and the optimization of industrial structure is conducive to weakening the initial inhibitory effect of environmental regulation. In addition, the analysis of regional heterogeneity showed that the impact of environmental regulation was stronger in the central and western regions, while the impact of industrial structure was stronger in the eastern and western regions. The conclusions of this study can help to expand the understanding of the relationship between environmental regulation and industrial structure on energy efficiency, provide policy enlightenment for the realization of green development and high-quality development, and provide Chinese examples and experiences for developing countries to improve energy efficiency.

## 1 Introduction

In recent years, extreme climate events have occurred frequently around the world, and the crisis of environmental pollution and resource depletion has been increasing. Behind all these phenomena is the excessive abuse of human resources. To address this situation, 178 countries signed the Paris Agreement in 2016, which promised to control the global average temperature increase within 2 degrees Celsius. Moreover, at the 26th United Nations Climate Conference held in 2021, it was once again emphasized that global net zero emissions should be ensured by the middle of this century, and global warming should be controlled at 1.5°C [1]. How to fundamentally change the mode of economic development, improve energy efficiency, reduce carbon emissions, and achieve the goal of carbon neutrality is an important proposition that all countries urgently need to solve after the COVID-19 outbreak [2]. As one of the signatories of the Paris Agreement, China has committed to achieving a carbon peak by 2030 and carbon neutrality by 2060, making its own contributions to protecting the environment.

China’s rapid economic development in the past 40 years has been achieved by adopting a rugged development model, which has been accompanied by considerable energy pressure and environmental pollution problems. According to the 2020 BP Statistical Yearbook of World Energy, China’s energy consumption accounted for 26.1% of the world’s total energy consumption in 2020, ranking first in the world for many years. A large amount of energy consumption combined with low energy efficiency leads to a large amount of sulfur dioxide, carbon dioxide, nitrogen dioxide and other polluting gas emissions, which exacerbates China’s domestic air pollution problem. According to the 2018 China Ecological and Environmental Status Bulletin issued by the Ministry of Environmental Protection, the area of acid rain in China in 2018 was approximately 530,000 square kilometers, accounting for 5.5% of the country’s total land area. After Europe and North America, China has become the third largest area of acid rain. In view of the current energy pressure and environmental pollution, the Chinese government has put forward the goal of transforming from high-speed development to high-quality development, and the report of the 20th National Congress of the Communist Party of China noted that "ecological and environmental problems, in the final analysis, are caused by excessive development, extensive utilization, luxury and waste of resources. Through green transformation and development, we must grasp the source of resource utilization." Therefore, comprehensively improving the efficiency of energy utilization has become an important way for China to change its development mode and achieve green transformation.

To improve energy efficiency, the Chinese government has issued a series of energy policies and environmental laws and regulations to promote efficient energy emission reduction [3]. However, different scholars hold different views on the specific effects of these government policies. Based on the "cost effect", traditional schools believe that environmental regulation will crowd out production costs, thus reducing energy efficiency. However, based on the "Porter hypothesis", other scholars believe that the government, as the maker of environmental regulation, will promote the improvement of regional environmental pollution whether it adopts command-type or market-type environmental regulation [4]. Although there has been a large amount of literature on the relationship between environmental regulation and energy efficiency, the relationship between environmental regulation and energy efficiency is still uncertain.

In addition, China’s industrial structure varies from region to region, and the industrial structure affects the scale and efficiency of energy use, as well as the effectiveness of government policies and regulations [5]. For example, adjusting the industrial structure will improve the problems of structural convergence, overcapacity, cutthroat competition and resource waste among enterprises, thus improving regional energy efficiency, and the top-down regulations implemented by the government will have different effects on different types of enterprises [6, 7]. Reviewing the literature, although there are a large number of separate studies on the impact of environmental regulation and industrial structure on energy efficiency, few studies have evaluated these three factors under the same framework. To address these problems, this paper studies the panel data of 30 provinces in China from 2005 to 2022 to explore the complex relationships among environmental regulation, industrial structure and energy efficiency.

This study aims to address the above research gap and contributes to the literature in two aspects. (1) From the perspective of reviewing the literature on environmental regulation, this paper analyzes the impact of environmental regulation on energy efficiency from the perspective of resource taxes for the first time and deeply analyzes the impact of resource taxes on energy efficiency. (2) Based on the requirements of industrial upgrading, this paper analyses the impact of industrial structure on energy efficiency and introduces the interaction term between environmental regulation and industrial structure to analyze the synergistic effect between environmental regulation and industrial structure. This analysis shows that maintaining the synergistic development of environmental regulation and industrial structure is highly important for promoting the development of clean energy and achieving energy conservation and emission reduction in China and other developing countries.

The remainder of the paper is organized as follows: The second section reviews the relevant literature and proposes the research hypotheses of this paper. The third section explains the empirical model and describes the data. The fourth section presents and describes the empirical results. The final section concludes and provides policy recommendations.

## 2 Literature review and research hypothesis

Based on the existing research, this section first discusses the impact of environmental regulation on energy efficiency and tries to clarify the relationship between the two. Then, we discuss the relationship between industrial structure and energy efficiency and determine whether upgrading the industrial structure can improve energy efficiency. Finally, on the basis of industrial structure, the moderating effect of environmental regulation on energy efficiency is further evaluated.

### 2.1 Environmental regulation and energy efficiency

In recent years, China’s economic growth has been slowing, and the downward pressure on the economy has been increasing. The once extensive economic development model can no longer match the current concept of high-quality development. Only through the efficient utilization of energy can we effectively promote the early realization of economic transformation and upgrading. As a means to restrict the pollution emissions of enterprises and enhance their awareness of environmental protection, environmental regulation can effectively improve the energy utilization rate of polluting industries and ultimately promote the energy revolution of the whole industry. When the concept of environmental regulation was first established, academia believed that environmental regulation would lead to an increase in the production costs of enterprises, thus reducing their competitiveness [8, 9]. However, Porter later proposed the famous "Porter hypothesis", in which he believed that strict environmental regulation can stimulate technological innovation, thus reducing production costs and gaining competitive advantages, and may also improve industrial productivity [10]. Therefore, the existing research on the relationship between environmental regulation and energy efficiency has not reached a unified conclusion. Most of the research results of early scholars support the "cost theory", and they believe that the government’s environmental control increases the cost of pollution control, thus reducing the energy efficiency of regional enterprises [11, 12]. However, in recent years, an increasing number of scholars have confirmed the "Porter hypothesis" in various aspects. In terms of research methods, such as system generalized method of moments (GMM), threshold model, SBM model, Tobit model and other methods and models [1, 2, 4, 13], or in terms of research perspective, whether measuring environmental regulation by policy means such as sulfur dioxide trading emission system (TES) and demonstration construction of new energy cities, whether it is measured by China’s environmental pollution liability insurance as an economic means or by China’s new Environmental Protection Law implemented in 2015 as a regulatory means, it shows that the government’s environmental protection measures in terms of policy, economy and regulations can significantly improve energy efficiency and reduce energy intensity [14–17]. Through the combination of related research, it can be seen that although scholars have measured environmental regulation from various angles and achieved many results, few scholars have measured the impact of the government on energy efficiency through this most direct means from the perspective of government taxation.

Moreover, with further research, several scholars have found that the relationship between environmental regulation and energy efficiency cannot be simply explained by a linear relationship and that there may be a nonlinear relationship between them. Wu and Wang [18] calculated the energy efficiency of 30 provinces in China by using the SBM method and found that there was an inverted U-shaped relationship between environmental regulation and energy efficiency. In addition, Chu and Wang [19], by dividing environmental regulation into command-and-control and market incentive types, found that the impact of command-and-control environmental regulation on energy efficiency exhibited an inverted U shape, while that of market incentive environmental regulation exhibited a U shape. However, compared with the inverted U-shaped relationship, the U-shaped relationship is more consistent with the analysis of the "cost effect" and the "Porter hypothesis". In the early stage of environmental regulation, regional enterprises will be more affected by the "cost effect", but with the development of enterprises and the deepening of government regulation, regional enterprises will shift to the "Porter hypothesis". For example, Hong et al. [20] divided environmental regulation into command-and-control and market-oriented regulation; however, their research showed that there was a U-shaped relationship between those two types and energy efficiency. In addition, Ren and Wu [21] used the GML index to measure energy efficiency and concluded that the impact of environmental regulation on energy efficiency exhibited a U-shaped relationship. Therefore, through the existing research, it can be found that there is still a large controversy about the relationship between the two, but it seems that the U-shaped relationship has gradually become mainstream, and this paper also tries to prove that there is a U-shaped relationship between the two to further enhance its theoretical validity.

There is a U-shaped relationship between environmental regulation and energy efficiency because environmental regulation affects the allocation of factors in the production process of enterprises, and energy, as a production factor, is inevitably affected by environmental regulation. Therefore, in the early stage of environmental regulation, improving relevant standards will increase the corresponding costs incurred by enterprises; for example, enterprises may be forced to pay pollution taxes or institute pollution prevention and control measures, thus increasing their production costs. However, an increase in production costs will crowd out the capital investment of enterprises in other aspects, especially investment in technological innovation [22]. Therefore, the "cost effect" will lead to a change in the capital investment structure of enterprises and reduce the relevant support for technological innovation [23]. Without the technological upgrading promoted by technological innovation, energy utilization efficiency may be reduced. Therefore, in the early stage of environmental regulation, environmental regulation leads to a reduction in the energy utilization efficiency of enterprises.

However, according to the Porter hypothesis, with the continuous increase in environmental regulation intensity, the "forced mechanism" of environmental regulation will in turn promote enterprise innovation. When the intensity of environmental regulation continues to increase, the increase in environmental costs will encourage enterprises to increase R&D investment related to energy efficiency to ultimately realize its positive effect on energy efficiency [24]. Understanding and following trends are the instincts of enterprises in response to market changes, and innovation is the core for enterprises seeking to survive in the market and improve market competitiveness. Therefore, when facing greater pressure from environmental regulation, enterprises will eventually choose to upgrade production technology to achieve high-quality production in the market [25]. Therefore, to some extent, strengthening environmental regulation will force enterprises to effectively allocate resources from a long-term perspective, that is, to increase technology research and development and related investment in energy utilization to achieve long-term energy efficiency improvement [26]. Based on the above analysis, this paper proposes the following hypothesis:

**H1:** Environmental regulation inhibits the improvement of energy efficiency in the initial stage but promotes the improvement of energy efficiency after a specified threshold is reached; that is, the relationship between environmental regulation and energy efficiency gradually follows a "U" shape.

### 2.2 Industrial structure and energy efficiency

As the link between production activities and energy efficiency, the industrial structure determines the conversion efficiency between the input and output of production factors [27]. By adjusting the industrial structure, the maximum output can be achieved under the condition that the total output remains unchanged to achieve the goals of energy conservation and emission reduction without reducing production [28]. However, the earliest academic discussion on the relationship between industrial structure and energy efficiency can be traced back to the structural dividend hypothesis proposed by Lewis in 1954 [29]. Since then, many scholars at home and abroad have begun to study this topic. However, there are still different opinions on whether the industrial structure can promote the improvement of regional energy efficiency and what type of corresponding relationship exists between them. Most scholars believe that adjusting the industrial structure has a positive effect on energy efficiency [30–33]; however, other scholars have found that a change in the industrial structure has no obvious impact on energy efficiency or even has the opposite effect [34, 35]. However, some scholars believe that there is a complex nonlinear relationship between the two [36, 37]. For this reason, although there are many studies on the relationship between industrial structure and energy efficiency, there is no unified conclusion on this topic, so this paper tries to make some contributions to understanding the relationship between the two.

The literature on the adjustment of industrial structure has mainly analyzed the adjustment of industrial institutions from the perspective of industrial structure optimization and usually measures the optimization of industrial structure by the ratio of secondary/tertiary industry to GDP. For example, Zou et al. [38] used the ratio of the added value of secondary industry and tertiary industry to GDP to measure the industrial structure. In production activities, the energy consumption of secondary industry is often greater than that of tertiary industry, so a higher proportion of secondary industry will lead to more energy input; thus, promoting the transformation from secondary industry to tertiary industry will improve the energy efficiency of the region [39]. As the "resource regulator" of economic input and output in each region, the adjustment of the industrial structure drives the corresponding structural adjustment of the product structure, market structure, organizational structure and other aspects and subsequently affects energy efficiency through the production mode and internal structure. Theoretically, the greater the proportion of secondary industry in a region is, the lower its energy efficiency will be because the industries in secondary industry, especially heavy industry, not only consume a large amount of energy but also lead to low energy efficiency due to their rough consumption mode. Compared with those in secondary industry, the service industry, the financial industry and other nonmaterial production industries in tertiary industry can generate larger profits with lower energy consumption [40]. Therefore, optimizing the industrial structure and changing rough economic development are important means of improving energy efficiency. Based on the above analysis, this paper proposes the following hypothesis:

**H2:** Industrial structure optimization is conducive to improving energy efficiency.

### 2.3 Regulatory effect of the industrial structure

The intensity of environmental regulation has different effects on different industries. Compared with light industries, heavy industries are more affected by government environmental control. Therefore, the industrial structure has a significant economic effect on regulating the relationship between environmental regulation and energy efficiency, but existing research does not include these three factors, and more studies consider more technological effects or innovation effects when analyzing the regulating effect or influencing path of environmental regulation on energy efficiency [24, 41, 42]. Compared with environmental regulation and energy efficiency, the regulatory effect of industrial structure is less considered, and the effect of industrial structure is considered more than other factors affecting energy efficiency, such as the regulatory effect or intermediary effect of industrial structure between digital finance and energy efficiency, financial technology and energy efficiency, and resource endowment and energy efficiency [43–45]. However, in recent years, with the significant role of industrial structure in improving energy efficiency, a few scholars have also begun to consider the regulatory role of industrial structure between environmental regulation and energy efficiency [46].

In the face of strict environmental standards, enterprises with poor technical levels often need to pay more, which will reduce their investment in their own technological research and development, resulting in low energy efficiency [47]. The optimization of industrial structure is often accompanied by the elimination of enterprises with inefficient production capacity and the transformation of enterprises to tertiary industries, such as financial services. This process will improve the average technical level of the remaining enterprises so that they can better cope with a series of environmental regulation measures promulgated by the government. In addition, the optimization of industrial structure means that enterprises with low technology levels will be gradually eliminated or removed from the region so that the spare production factors in the region will be reused by more efficient enterprises. Therefore, with the continuous optimization of industrial structure, the inhibitory effect of environmental regulation on energy efficiency will continuously weaken in the early stage. Based on the above analysis, this paper proposes the following hypothesis:

**H3:** Industrial structure optimization is conducive to weakening the inhibitory effect of initial environmental regulation on energy efficiency.

## 3 Research design

### 3.1 Model setting

Based on the above theoretical analysis, this paper empirically studies the impact of environmental regulation and the industrial structure on energy efficiency. Based on the empirical research of Dzwigol et al. (2023), Wei et al. (2022), and WU et al. (2020), the quadratic term of environmental regulation is added to the model [48–50]. To further verify the nonlinear effect between environmental regulation and energy efficiency, Hypothesis H1 is verified:

$$EE_{\mathrm{it}}=\alpha_{0}+\alpha_{1}ER_{\mathrm{it}}+\alpha_{2}E{R_{\mathrm{it}}}^{2}+\alpha_{3}TL_{\mathrm{it}}+\alpha_{4}INV_{\mathrm{it}}+\alpha_{5}PGDP_{\mathrm{it}}+\alpha_{6}UL_{\mathrm{it}}+\mu_{i}+\psi_{t}+\varepsilon_{\mathrm{it}}$$ (1)

To test Hypothesis 2, this paper constructs the following regression model:

$$EE_{\mathrm{it}}=\beta_{0}{+\beta }_{1}IS_{\mathrm{it}}+\beta_{2}TL_{\mathrm{it}}+\beta_{3}INV_{it}+\beta_{4}PGDP_{it}+\beta_{5}UL_{it}+\mu_{i}+\psi_{t}+\varepsilon_{it}$$ (2)

Moreover, to further investigate whether there is a synergistic effect between environmental regulation and industrial structure, the interaction term between environmental regulation and industrial structure is added to the model to explore whether industrial structure can regulate environmental regulation to affect energy efficiency, and the following adjustment model is obtained to verify Hypothesis H3:

$$EE_{\mathrm{it}}=\gamma_{0}{+\gamma }_{1}ER_{\mathrm{it}}+\gamma_{2}{ER}_{\mathrm{it}}\times IS_{\mathrm{it}}+\gamma_{3}TL_{\mathrm{it}}+\gamma_{4}INV_{it}+\gamma_{5}PGDP_{it}+\gamma_{6}UL_{it}+\mu_{i}+\psi_{t}+\varepsilon_{it}$$ (3)

In Eqs (1)–(3), i and t represent the province and year, respectively; EE represents the explanatory variable and energy efficiency; ER represents environmental regulation; IS represents the industrial structure; TL represents the technology level; INV represents the capital factor level; PGDP represents the economic development level; UL represents the urbanization level; α_i_, β_i_ and γ_i_ represent the coefficients of each variable; μ_i_ and ψ_i_ represent the province fixed and year fixed, respectively; and ε represents the random perturbation term.

### 3.2 Variables and data sources

This paper selects the panel data of 30 provinces, cities, and autonomous regions in China (based on the integrity and availability of data, excluding Hong Kong, Macao, Taiwan, and Tibet) from 2005 to 2021 as the basis of empirical analysis. The data used mainly come from the National Bureau of Statistics of China, the "China Statistical Yearbook", the "China Energy Statistical Yearbook", the "China Environmental Statistical Yearbook", the "China Environmental Yearbook", the China Environmental Statistical Yearbook, and the statistical yearbooks of various provinces. The carbon emission data are sourced from the China Carbon Accounting Database. The processing and description of the relevant data are as follows:

1. The explained variable. This study employs the single-factor approach of Chang et al. to measure energy efficiency [51]. Specifically, energy efficiency is determined by calculating the ratio of GDP to total energy consumption in each region. Unlike total factor energy efficiency, this technique more directly reflects a region’s energy costs associated with economic growth. Higher indices reflect increased energy efficiency, meaning more output per unit of energy consumed.
2. Explanatory variables. In environmental regulation (ER), the main function is to prevent and curb pollution from the source. Therefore, this article uses the government-imposed resource tax as a proxy for environmental regulation, with higher tax figures indicating a greater level of regulation. The industrial structure (IS) is characterized by high levels of pollution and energy consumption associated with secondary industry compared to tertiary industry. Inspired by Huang et al. [52], we apply the ratio of the added value of tertiary industry to the added value of secondary industry to represent the industrial structure. Larger values indicate a greater proportion of tertiary industry and a more balanced and complete industrial structure.
3. Control variables. In this paper, the WSR method is adopted; that is, the control variables are selected from the three levels of "physics-affairs-human affairs". 1) "Physical level" This paper draws on Yu and Tang and Sun et al. [53, 54] to select the technical level (TL), that is, the ratio of regional R&D expenditure to regional GDP. 2) At the " rational " level, this paper draws on Wen et al., Hou et al., Hou et al., and others to select the capital factor (INV), that is, the amount of investment in fixed assets [55, 56]. 3) At the level of "human reason", this paper draws on Zhu and Li, Kolosok et al. [57, 58] to select the level of economic development (PGDP) and Yv et al. and Li et al. [59, 60] to select the level of urbanization (UL), in which the level of economic development is expressed by per capita GDP and the level of urbanization is expressed by the ratio of the urban population to the permanent population at the end of the year.

### 3.3 Descriptive statistics of variables

The results in Table 1 depict the descriptive statistics of the main variables examined in this article. The average energy efficiency (EE) is 1.389, with a maximum of 5.778 and a minimum of 0.229. The standard deviation of EE is 0.843. This suggests a notable difference in the level of energy efficiency among the various regions examined. Additionally, the industrial structure has a low mean and a large standard error, whereas environmental regulation (ER) has both traits. Regarding the control variables, notable discrepancies exist in the levels of economic development (PGDP) and capital factors (INV) across the various provinces analyzed, while the differences in urbanization level (UL) and technology level (TL) are relatively minor.

**Table 1:** Descriptive statistics of the panel data examined in the study.

Variable	Definition	Units	N	mean	sd	max	min
EE	Energy efficiency	100 million yuan/ten thousand tons of standard coal	510	1.389	0.843	5.778	0.229
ER	Environmental regulation	100 billion yuan	510	0.031	0.055	0.494	0.000
IS	Industry structure	-	510	1.220	0.678	5.244	0.527
PGDP	Level of economic development	10000 RMB/person	510	4.462	2.951	18.753	0.522
UL	urbanization	-	510	0.558	0.140	0.896	0.269
TL	Skill level	-	510	0.016	0.011	0.066	0.002
INV	Level of capital factor	Trillion yuan	510	1.429	1.330	6.269	0.033

## 4 Empirical results

### 4.1 Regression analysis

This article initially explored the relationship between environmental regulations and energy efficiency using Model (1). Table 2, Column (1) shows that in the fixed effects regression, the coefficient of environmental regulation (ER) is significantly negative, with a p value < 0.01, while the coefficient of the squared term of environmental regulation (ER^2^) is significantly positive, with a p value < 0.05. These results suggest a curvilinear relationship linking environmental regulation to energy efficiency. Environmental regulation is negatively correlated with energy efficiency when its degree is low but positively correlated when the regulation is stringent, indicating a "U"-shaped relationship between the two that first inhibits and later enhances. When the government’s environmental regulation is low, local governments are more willing to pay lower environmental costs to ensure their own production than to invest much money in technology research and development; that is, following the "cost effect" reduces local energy efficiency [12]. However, as government control becomes increasingly stringent, inputs and outputs will be disproportionate when investing funds in production, and increasing technology research and development and improving their own use efficiency will bring greater benefits; thus, local governments will carry out technological innovation, thereby improving regional energy efficiency [61]. In addition, strict environmental regulations further improve a region’s energy efficiency by eliminating inefficient companies and optimizing the flow of resources in the region [62]. The results of this paper are also consistent with those of Wu [50] and Zhao [63], thus validating Hypothesis H1.

**Table 2:** Impact of environmental regulation and industrial structure on energy efficiency.

Variable	(1)	(2)	(3)
ER	-2.337***		-3.369***
	(-0.420)		(0.664)
ER^2^	5.994**		
	(2.402)		
IS		0.326***	
		(0.058)	
ER×IS			0.002***
			(0.001)
PGDP	0.249***	0.246***	0.249***
	(0.136)	(0.014)	(0.014)
UL	-0.820*	0.261	-0.660
	(0.496)	(0.574)	(0.497)
TL	-4.998	-1.199	-4.044
	(4.325)	(4.344)	(4.306)
INV	0.123***	0.136***	0.117***
	(0.018)	(0.018)	(0.018)
_cons	0.721***	-0.153	0.636***
	(0.248)	(0.314)	(0.249)
Prov FE	YES	YES	YES
Year FE	YES	YES	YES
N	510	510	510
R^2^	0.906	0.903	0.907

Notes: The 1%, 5% and 10% levels of significance are indicated by ***, ** and *, respectively. Standard errors are in parentheses.

To further test Hypothesis H2, Model (2) is used to investigate the impact of the industrial structure on energy efficiency. As shown in Column (2) of Table 2, the coefficient of industrial structure (IS) is 0.326, which is significant at the level of 1%, indicating that there is a positive correlation between industrial structure and energy efficiency [64]; that is, every 1 percentage point optimization of industrial structure will promote an improvement in energy efficiency of 0.33%. The optimization of industrial structure is the transformation and upgrading of regional secondary industry to tertiary industry, and tertiary industry is often a green industry with a high input‒output ratio [65]. Therefore, the optimization of the industrial structure can positively promote the improvement of energy efficiency, and Hypothesis 2 is verified.

In a previous study, the analysis examined how industrial structure moderates the impact of environmental regulation on energy efficiency. Model (3) was constructed to verify the moderating effect hypothesis [66], and the results presented in Column (3) of Table 2 indicate that the coefficient of environmental regulation (ER) is significantly negative. Simultaneously, the coefficient of the interaction between environmental regulation and industrial structure (ER × IS) is significantly positive. This signifies that the optimization of the industrial structure regionally reduces the negative effect of initial environmental regulations on energy efficiency. Moreover, environmental regulations, in conjunction with industrial structure optimization, promote an increase in energy efficiency. Therefore, these findings support Hypothesis 3 of this study.

### 4.2 Regional heterogeneity testing

In fact, due to the different government policy goals and factor endowments, whether considering energy efficiency, environmental regulation or industrial structure, there are obvious heterogeneity characteristics in the regional distribution. For example, Zhang et al. (2022), Liu et al. (2023), and Qin et al. (2021) believe that environmental regulation has different impacts on different regions [67–69]. Therefore, this paper further conducts a regional heterogeneity analysis, and the results are shown in Table 3. The results reveal that there is a U-shaped relationship between environmental regulation and energy efficiency in the central and western regions, but this relationship is not significant in the eastern region. The reason for this difference may be that, compared with those in the central and western regions, the eastern region comprises mostly developed cities, the proportion of secondary industry in its industrial structure is small, and the proportion of heavy industry is even smaller. Therefore, environmental regulation does not play a positive role in promoting energy efficiency [70]. However, the central and western regions are concentrated industrial areas, and the government’s environmental protection measures can urge enterprises to improve their own technology to cope with increasingly strict environmental standards. In contrast, the industrial structure plays a positive role in promoting the eastern and western regions, while its role in the central region is not obvious. This may be because the industrial structure of the western region generally relies on the unique path of transforming its own natural resources and location advantages into social productivity; thus, it focuses on improving its own technological innovation in energy utilization when optimizing its industrial structure and realizing the improvement of energy efficiency by improving technology [71]. In addition, the eastern region relies mainly on information technology and modern services to optimize the industrial structure, which can greatly promote the efficiency of energy use. However, as a transfer zone for heavily polluting industries in the eastern region, the optimization of the industrial structure in the central region does not have an obvious effect on energy efficiency and may even have an inhibitory effect [72]. Therefore, the optimization of the industrial structure can promote the improvement of energy efficiency in the eastern and western regions but has no obvious effect on the central region.

**Table 3:** Impact of environmental regulation and industrial structure on energy efficiency in different regions.

Variable	east	mid	west
ER	-6.191**	-3.365**	-3.136**
	(3.045)	(1.392)	(-4.06)
ER^2^	-41.758	9.080***	36.092***
	(107.656)	(2.394)	(6.360)
IS	0.292***	-0.274	0.446***
	(0.087)	(0.084)	(0.102)
ER×IS	0.004***	0.001	-0.003**
	(0.001)	(0.001)	(0.001)
PGDP	0.239***	0.069**	0.313***
	(0.018)	(0.030)	(0.035)
UL	-1.496	0.078	5.129***
	(1.007)	(0.912)	(1.051)
TL	-9.589*	-2.165	-5.927
	(5.692)	(8.204)	(6.831)
INV	0.105***	0.213***	0.405***
	(0.034)	(0.030)	(0.030)
_cons	1.018	0.740*	-2.063***
	(5.27)	(0.375)	(0.413)
Prov FE	YES	YES	YES
Year FE	YES	YES	YES
N	204	153	153
R^2^	0.945	0.970	0.956

Notes: The 1%, 5% and 10% levels of significance are indicated by ***, ** and *, respectively. Standard errors are in parentheses.

### 4.3 Robustness test

To further exclude the influence of other factors on energy efficiency and ensure that the conclusions drawn in this paper are related to environmental regulation and the industrial structure, it is necessary to conduct a robustness test. OFDI will have an important impact on the technical level and production capacity of the region and subsequently on the energy efficiency of the region [73, 74]. Therefore, in view of the impact that the differences in OFDI among different provinces may have on the empirical results, this paper sets corresponding control variables for the OFDI of different provinces. The new control variables are added to the benchmark regression model to conduct a robustness test. Table 4 reports the corresponding regression results. The test results are consistent with the empirical results, with only slight coefficient differences. Therefore, the results support the robustness of the conclusions of this paper and further verify Hypothesis 1, Hypothesis 2 and Hypothesis 3.

**Table 4:** Robustness test: Adding control variables.

Variable	(1)	(2)	(3)
ER	-2.653***		-3.427***
	(0.414)		(0.650)
ER^2^	6.860***		
	(2.346)		
IS		0.299***	
		(0.059)	
ER×IS			0.002***
			(0.001)
PGDP	0.248***	0.245***	0.249***
	(0.013)	(0.014)	(0.132)
UL	-0.529	0.244	-0.399
	(0.486)	(0.570)	(0.489)
TL	-9.738**	-3.335	-8.416*
	(4.318)	(4.398)	(4.310)
INV	0.087***	0.117***	0.083***
	(0.019)	(0.019)	(0.019)
FDI	0.001***	0.001**	0.001***
	(0.001)	(0.001)	(0.001)
_cons	0.613**	-0.109	0.539**
	(0.243)	(0.312)	(0.244)
Prov FE	YES	YES	YES
Year FE	YES	YES	YES
N	510	510	510
R^2^	0.911	0.904	0.911

Notes: The 1%, 5% and 10% levels of significance are indicated by ***, ** and *, respectively. Standard errors are in parentheses.

Moreover, due to the existence of omitted variables and their correlation with other explanatory variables, such as the strong correlation between the resource consumption structure and the industrial structure, resource endowment and energy efficiency [75–77], the residual term will be correlated with other explanatory variables due to the existence of these situations. As a result, the empirical model of this paper has several endogeneity problems. Therefore, in view of the relevant endogeneity problems, this paper uses the lagged term of explanatory variables as an instrumental variable and adopts the one-period lagged explanatory variable and instrumental variable method to solve the endogeneity problems of this paper [78, 79], which can not only consider the influence of other factors but also reduce model error. Column (1) of Table 5 reports the impact of environmental regulation in period t on energy efficiency in period t+1, and the results show that there is still a U-shaped relationship between environmental regulation and energy efficiency. Moreover, in the validity test of the instrumental variables, the F-statistic of the weak correlation test is 150.118, indicating that the instrumental variables in this paper are strongly correlated. In addition, in the overidentification test, the P values of Basmann and Sargan are greater than 0.5, so the instrumental variables constructed in this paper meet the requirements of correlation and exogeneity. In Column (3) of Table 5, the results of the second-stage regression considering endogeneity show that the coefficients of environmental regulation and industrial structure and their significance support the benchmark results.

**Table 5:** Robustness test: Lagged regression of explanatory variables and least square regression of instrumental variables.

	Explanation variable lag regression	Two-stage least squares regression for instrumental variables	
	(1)EE	(2) Stage 1: ER	(3) Stage 2: EE
ER	-6.886***		-11.228***
	(1.304)		(2.410)
ER^2^	8.624***		29.971***
	(2.228)		(4.954)
IS	0.283***		0.292***
	(0.059)		(0.042)
ER×IS	2.030		0.004**
	(0.884)		(0.002)
IV		0.496***	
		(0.041)	
_cons	-0.255	0.008***	0.667***
	(0.297)	(0.003)	(0.105)
Prov Fe	YES	YES	YES
Year Fe	YES	YES	YES
N	510	510	510
R^2^	0.915	0.970	0.810

Notes: The 1%, 5% and 10% levels of significance are indicated by ***, ** and *, respectively. Standard errors are in parentheses.

## 5 Conclusion and policy implications

Given the escalation of global warming and environmental challenges, energy has become the primary focus of the United Nations Climate Change Conference. Most environmental and climate problems are intricately linked to energy efficiency. Therefore, enhancing energy efficiency has become a pressing issue worldwide. China’s drive toward high-quality development, as proposed by the government in 2017, and its "dual carbon" goal, proposed by the United Nations General Assembly in 2020, necessitate upgrading industrial technology and optimizing energy utilization efficiency. Governmental environmental regulations and industrial structure optimization are key ways to improve energy efficiency and achieve high-quality development goals. Based on an analysis of the pertinent literature, this paper explores the theoretical underpinnings of the relationships between environmental regulation, industrial structure, and energy efficiency. Then, using fixed-effects panel data from 30 provinces and cities in China ranging from 2005 to 2021, this paper examines the relationships between environmental regulation, industrial structure, and energy efficiency. The test results reveal a U-shaped correlation between environmental regulations and energy efficiency in China. When environmental regulations are low, they can even decrease enterprises’ energy efficiency. However, when the administration fortifies and rationalizes environmental regulations progressively, it can substantially enhance enterprise production and improve energy efficiency. The industrial structure regression results demonstrate a significant association with energy efficiency. Moreover, industrial structure optimization has a "structural dividend" effect, further boosting energy efficiency improvement. The regression results for the industrial structure show a positive correlation with energy efficiency, and the optimization of the industrial structure has a "structural dividend" effect, which can promote the improvement of energy efficiency. A deeper understanding of the impact of environmental regulation and the industrial structure on energy efficiency reflects the ability of industrial structure optimization to mitigate the low valley effect of environmental regulation, which weakens its inhibitory impact on environmental regulation. Overall, the interaction between industrial structure and environmental regulation enhances regional energy efficiency levels.

Due to differences in resource endowments between provinces, the impact of environmental regulations and industrial structure on energy efficiency may vary across regions. The impact of environmental regulations is mainly associated with secondary industry resource consumption. Therefore, government regulations have proven to be less effective at enhancing energy efficiency, given the advancements in clean energy and recent upgrades in the eastern province. The central and western provinces rely on their endowed resources for development, and under a government policy that seeks to protect the environment, government interventions can increase energy efficiency. From the perspective of industrial structure, improvements in energy efficiency in the eastern region can be attributed to the optimization of industrial structure. The shift toward a low-carbon industry has significantly improved energy efficiency in the region.

The conclusions of this article not only enhance the understanding of the relationships among environmental regulations, industrial institutions, and energy efficiency but also contain important policy implications.

1. Further improvement in resource tax legislation is necessary to surpass the threshold at which prior environmental regulations impact energy efficiency. Additionally, the government needs to avoid excessive resource taxation, which hinders enterprise energy utilization to enable resource taxes to genuinely boost resource utilization efficiency. Enhancements to laws and policies should promote enterprises’ awareness of how to utilize resources effectively and dissuade the increased utilization of resources by firms without corresponding technological improvements. Resource tax targets and government measures for taxation based on quantity and quota require consistent improvement.
2. China needs to further promote industrial structure optimization and firmly implement high-quality development. China needs to vigorously promote the technological upgrading of enterprises to achieve the continuous upgrading of China’s secondary industry. The market’s business exit strategy should be enhanced, and inefficient companies should be eliminated to facilitate the efficient flow of resources. Simultaneously, industry-specific policies should be developed and reinforced for the consistent development of inefficient, high-energy tertiary industries. Finally, China must increase its investment in technological research and development to promote the upgrading of energy-efficient practices within secondary industry to ultimately enhance China’s energy efficiency.
3. To avoid adopting a "one size fits all" approach, it is crucial to adapt to local conditions and enforce diverse environmental regulatory policies based on each region’s particular needs. China’s vast land area and varied levels of development across regions highlight local governments’ essential role in environmental regulation. To enable effective regulations, the central government must provide guidelines and delegate more power to local authorities for successful implementation. Furthermore, local administrations should generate environment-specific regulations according to their domestic business requirements, establish harmonizing measures for resource tax collection, and create sound pollution monitoring investments and other policies to promote energy efficiency and local environmental conditions.

Although the results of this paper are instructive to some extent, there are still some limitations. First, this paper considers only the macro impact of resource taxes set up by local governments on regional energy efficiency and does not consider the impact at the industry or enterprise level. Future studies can consider using existing data to explore this aspect. In addition, although this paper considers the moderating effect of industrial structure on the impact of environmental regulation on energy efficiency, it does not further analyze the underlying mechanism involved. Future research can explore the relevant mechanisms to better help the government implement appropriate environmental policies.

## Supporting information

S1 Data (DOCX)
