# Supplementary material for: Environmental regulation, industrial structure and energy efficiency: Evidence from 30 provinces in China
Source: PLoS One. 2024 May 20;19(5):e0299731. doi: 10.1371/journal.pone.0299731 (PMC11104590; doi:10.1371/journal.pone.0299731)
Supplement: S1 Data — (DOCX) [file pone.0299731.s001.docx]

| prv | year | ee | er | is | pgdp | ul | tl | inv | fdi |
| --- | --- | --- | --- | --- | --- | --- | --- | --- | --- |
| Beijing | 2005 | 1.294798 | 0.29 | 2.702894 | 4.7182 | 0.836151 | 0.053442 | 0.282723 | 35.26 |
| Beijing | 2006 | 1.420535 | 0.31 | 3.005502 | 5.3438 | 0.843223 | 0.051628 | 0.329638 | 45.52 |
| Beijing | 2007 | 1.658779 | 0.31 | 3.278694 | 6.3629 | 0.844869 | 0.048477 | 0.39072 | 50.66 |
| Beijing | 2008 | 1.867055 | 0.36 | 3.631258 | 6.8541 | 0.849238 | 0.046584 | 0.381473 | 60.82 |
| Beijing | 2009 | 1.963505 | 0.42 | 3.671868 | 7.1059 | 0.85 | 0.051826 | 0.461692 | 61.21 |
| Beijing | 2010 | 2.15184 | 0.37 | 3.590393 | 7.8307 | 0.859327 | 0.054918 | 0.540295 | 63.64 |
| Beijing | 2011 | 2.457158 | 0.34 | 3.786097 | 8.6246 | 0.862154 | 0.054489 | 0.557893 | 70.54 |
| Beijing | 2012 | 2.650535 | 0.8 | 3.895306 | 9.2758 | 0.862849 | 0.055896 | 0.611237 | 80.416 |
| Beijing | 2013 | 3.143208 | 0.82 | 4.03198 | 10.0569 | 0.864 | 0.056069 | 0.684706 | 85.2418 |
| Beijing | 2014 | 3.356059 | 0.78 | 4.135777 | 10.6732 | 0.865039 | 0.055343 | 0.692423 | 90.4085 |
| Beijing | 2015 | 4.0735 | 0.91 | 4.574619 | 11.3692 | 0.867002 | 0.055854 | 0.749599 | 129.964 |
| Beijing | 2016 | 3.909383 | 0.75 | 4.767821 | 12.3391 | 0.867426 | 0.054901 | 0.794389 | 130.286 |
| Beijing | 2017 | 4.215999 | 1.08 | 4.893987 | 13.6172 | 0.869189 | 0.052863 | 0.837044 | 243.291 |
| Beijing | 2018 | 4.553783 | 27.23 | 5.022109 | 15.0962 | 0.870894 | 0.056509 | 0.791007 | 173.109 |
| Beijing | 2019 | 4.81591 | 32.63 | 5.23404 | 16.1776 | 0.873516 | 0.06564 | 0.771231 | 142.13 |
| Beijing | 2020 | 5.315405 | 29.64 | 5.24401 | 16.4158 | 0.875286 | 0.06473 | 0.788199 | 141.044 |
| Beijing | 2021 | 5.778124 | 31.28 | 4.539884 | 18.7526 | 0.874829 | 0.064059 | 0.82682 | 155.616 |
| Tianjin | 2005 | 0.7733 | 0.47 | 0.868261 | 3.0567 | 0.750719 | 0.022985 | 0.149514 | 33.29 |
| Tianjin | 2006 | 0.78624 | 0.5 | 0.872336 | 3.3411 | 0.757209 | 0.026906 | 0.182052 | 41.31 |
| Tianjin | 2007 | 0.841301 | 0.58 | 0.907563 | 3.7976 | 0.763229 | 0.027583 | 0.235315 | 52.78 |
| Tianjin | 2008 | 0.966218 | 0.76 | 0.904651 | 4.5242 | 0.772109 | 0.030044 | 0.338979 | 74.2 |
| Tianjin | 2009 | 0.971998 | 0.69 | 0.990245 | 4.7497 | 0.78013 | 0.031263 | 0.47382 | 90.2 |
| Tianjin | 2010 | 1.001866 | 0.56 | 1.055097 | 5.4053 | 0.795997 | 0.033612 | 0.62781 | 108.49 |
| Tianjin | 2011 | 1.067652 | 0.73 | 1.122168 | 6.1458 | 0.804623 | 0.036709 | 0.706767 | 130.56 |
| Tianjin | 2012 | 1.101729 | 2.67 | 1.151693 | 6.6517 | 0.815675 | 0.039865 | 0.793478 | 150.163 |
| Tianjin | 2013 | 1.261813 | 3.62 | 1.221574 | 7.1345 | 0.822695 | 0.024947 | 0.913025 | 168.29 |
| Tianjin | 2014 | 1.306387 | 2.76 | 1.271 | 7.496 | 0.825752 | 0.043672 | 1.05182 | 188.668 |
| Tianjin | 2015 | 1.307789 | 2.1 | 1.387117 | 7.5868 | 0.829048 | 0.046896 | 1.1832 | 211.344 |
| Tianjin | 2016 | 1.420797 | 1.69 | 1.589011 | 7.9647 | 0.832987 | 0.046815 | 1.27794 | 101.005 |
| Tianjin | 2017 | 1.589709 | 2.56 | 1.690914 | 8.728 | 0.835461 | 0.036842 | 1.12889 | 106.078 |
| Tianjin | 2018 | 1.676019 | 11.34 | 1.727359 | 9.5689 | 0.839479 | 0.036848 | 1.06567 | 48.5104 |
| Tianjin | 2019 | 1.705558 | 12.82 | 1.803626 | 10.1557 | 0.843321 | 0.034506 | 1.20528 | 47.3161 |
| Tianjin | 2020 | 1.728401 | 11.56 | 1.809092 | 10.1068 | 0.847152 | 0.034623 | 1.24144 | 47.3536 |
| Tianjin | 2021 | 1.911491 | 11.7 | 1.718141 | 11.366 | 0.848507 | 0.036616 | 1.30102 | 53.89 |
| Hebei | 2005 | 0.442297 | 11.03 | 0.801232 | 1.2845 | 0.376879 | 0.006713 | 0.413969 | 19.13 |
| Hebei | 2006 | 0.460813 | 17.52 | 0.810851 | 1.4609 | 0.387649 | 0.007637 | 0.547024 | 20.14 |
| Hebei | 2007 | 0.515278 | 24.09 | 0.780648 | 1.7561 | 0.402564 | 0.007406 | 0.688468 | 24.16 |
| Hebei | 2008 | 0.583841 | 23.66 | 0.753678 | 2.0385 | 0.418944 | 0.007683 | 0.886656 | 34.19 |
| Hebei | 2009 | 0.602188 | 23.61 | 0.840363 | 2.1831 | 0.437447 | 0.008806 | 1.22698 | 35.98 |
| Hebei | 2010 | 0.653937 | 25.77 | 0.833481 | 2.5308 | 0.444954 | 0.008632 | 1.50834 | 38.31 |
| Hebei | 2011 | 0.724947 | 32.77 | 0.818101 | 2.9647 | 0.45589 | 0.009413 | 1.63893 | 46.81 |
| Hebei | 2012 | 0.762887 | 54.26 | 0.846525 | 3.1844 | 0.465987 | 0.010651 | 1.96613 | 58.0486 |
| Hebei | 2013 | 0.817802 | 56.96 | 0.889152 | 3.3346 | 0.480241 | 0.01162 | 2.31942 | 64.472 |
| Hebei | 2014 | 0.859779 | 54.68 | 0.920745 | 3.4507 | 0.49365 | 0.01242 | 2.66719 | 63.7196 |
| Hebei | 2015 | 0.325757 | 28.36 | 1.022475 | 3.5994 | 0.516678 | 0.013292 | 2.94483 | 61.775 |
| Hebei | 2016 | 0.905147 | 30.2 | 1.058951 | 3.8688 | 0.538712 | 0.013465 | 3.175 | 73.5388 |
| Hebei | 2017 | 0.37329 | 44.98 | 1.152982 | 4.1451 | 0.55743 | 0.014752 | 3.34068 | 84.8951 |
| Hebei | 2018 | 1.009619 | 48.03 | 1.259445 | 4.3808 | 0.573256 | 0.015378 | 3.54112 | 90.811 |
| Hebei | 2019 | 1.074777 | 56.13 | 1.348873 | 4.7036 | 0.587753 | 0.018137 | 3.77129 | 98.5 |
| Hebei | 2020 | 1.098551 | 53.06 | 1.334418 | 4.8302 | 0.60075 | 0.017615 | 3.89198 | 108.508 |
| Hebei | 2021 | 1.223339 | 74.2 | 1.223474 | 5.4181 | 0.611439 | 0.017579 | 4.00873 | 112.9 |
| Shanxi | 2005 | 0.319957 | 18.17 | 0.603515 | 1.2195 | 0.421162 | 0.006447 | 0.182658 | 2.75 |
| Shanxi | 2006 | 0.334341 | 20.4 | 0.592255 | 1.4008 | 0.429926 | 0.007701 | 0.225574 | 4.72 |
| Shanxi | 2007 | 0.380461 | 25.89 | 0.563473 | 1.7542 | 0.440318 | 0.008306 | 0.286146 | 13.43 |
| Shanxi | 2008 | 0.460785 | 31.74 | 0.560606 | 2.1234 | 0.451187 | 0.008667 | 0.353116 | 10.23 |
| Shanxi | 2009 | 0.458892 | 30.05 | 0.638688 | 2.0906 | 0.459877 | 0.011318 | 0.494316 | 4.93 |
| Shanxi | 2010 | 0.529741 | 32.65 | 0.568996 | 2.5434 | 0.480414 | 0.010097 | 0.60632 | 7.14 |

| Shanxi | 2011 | 0.594831 | 38.76 | 0.527051 | 3.0534 | 0.498035 | 0.010409 | 0.707306 | 20.73 |
| --- | --- | --- | --- | --- | --- | --- | --- | --- | --- |
| Shanxi | 2012 | 0.604228 | 43.78 | 0.611204 | 3.2864 | 0.513247 | 0.011324 | 0.886326 | 25.0379 |
| Shanxi | 2013 | 0.606595 | 51.65 | 0.688973 | 3.3848 | 0.528713 | 0.01293 | 1.10319 | 28.0667 |
| Shanxi | 2014 | 0.608913 | 58.9 | 0.780825 | 3.4248 | 0.543084 | 0.012584 | 1.23545 | 29.5186 |
| Shanxi | 2015 | 0.622019 | 143.18 | 1.128513 | 3.3593 | 0.558681 | 0.011194 | 1.40742 | 28.6985 |
| Shanxi | 2016 | 0.629619 | 140.35 | 1.19456 | 3.3972 | 0.572567 | 0.0111 | 1.4198 | 23.3242 |
| Shanxi | 2017 | 0.739712 | 272.69 | 1.074526 | 4.1242 | 0.58604 | 0.010232 | 0.604054 | 16.9049 |
| Shanxi | 2018 | 0.790044 | 325.17 | 1.151021 | 4.5517 | 0.598515 | 0.011016 | 0.638485 | 23.6171 |
| Shanxi | 2019 | 0.813155 | 383.03 | 1.161218 | 4.8469 | 0.612811 | 0.012446 | 0.697864 | 13.5904 |
| Shanxi | 2020 | 0.850124 | 358.73 | 1.163906 | 5.1051 | 0.625215 | 0.011836 | 0.771838 | 16.9058 |
| Shanxi | 2021 | 1.056665 | 493.51 | 0.864166 | 6.5625 | 0.634195 | 0.011014 | 0.838988 | 17 |
| Inner Mongolia | 2005 | 0.364542 | 6.64 | 1.129554 | 1.4695 | 0.47191 | 0.00332 | 0.26436 | 11.86 |
| Inner Mongolia | 2006 | 0.370901 | 12.78 | 1.047666 | 1.7275 | 0.486542 | 0.003965 | 0.336321 | 17.41 |
| Inner Mongolia | 2007 | 0.404385 | 16.68 | 1.074905 | 2.1334 | 0.501441 | 0.004684 | 0.437288 | 21.49 |
| Inner Mongolia | 2008 | 0.442713 | 22.06 | 1.089742 | 2.562 | 0.517185 | 0.005431 | 0.547541 | 26.51 |
| Inner Mongolia | 2009 | 0.463009 | 27.55 | 1.115706 | 2.8982 | 0.534174 | 0.00732 | 0.733679 | 29.84 |
| Inner Mongolia | 2010 | 0.4875 | 36.82 | 1.076334 | 3.3262 | 0.555016 | 0.007768 | 0.89265 | 33.85 |
| Inner Mongolia | 2011 | 0.504784 | 56.81 | 1.014936 | 3.8276 | 0.570445 | 0.009008 | 1.03652 | 38.38 |
| Inner Mongolia | 2012 | 0.529175 | 68.1 | 0.98017 | 4.2441 | 0.58401 | 0.009685 | 1.18757 | 41.7665 |
| Inner Mongolia | 2013 | 0.644317 | 70.64 | 1.01423 | 4.632 | 0.598371 | 0.010288 | 1.42174 | 48.4258 |
| Inner Mongolia | 2014 | 0.664054 | 72.69 | 1.056976 | 4.9585 | 0.609637 | 0.010043 | 1.75918 | 41.7182 |
| Inner Mongolia | 2015 | 0.689363 | 105.3 | 1.147965 | 5.2972 | 0.620902 | 0.01051 | 1.37022 | 33.6629 |
| Inner Mongolia | 2016 | 0.714102 | 118.16 | 1.175472 | 5.656 | 0.633826 | 0.010697 | 1.508 | 29.6672 |
| Inner Mongolia | 2017 | 0.753838 | 204.03 | 1.255299 | 6.1196 | 0.646116 | 0.00888 | 1.40132 | 31.4951 |
| Inner Mongolia | 2018 | 0.699705 | 238.49 | 1.27138 | 6.6491 | 0.655244 | 0.008005 | 1.00474 | 31.5869 |
| Inner Mongolia | 2019 | 0.679101 | 302.33 | 1.269551 | 7.117 | 0.664596 | 0.009359 | 1.07206 | 20.6105 |
| Inner Mongolia | 2020 | 0.636037 | 300.79 | 1.204525 | 7.164 | 0.67499 | 0.009335 | 1.05598 | 18.224 |
| Inner Mongolia | 2021 | 0.728674 | 385.12 | 0.903943 | 8.8137 | 0.682083 | 0.008296 | 1.15947 | 3.2 |
| Liaoning | 2005 | 0.533461 | 12.05 | 0.860217 | 1.721 | 0.587065 | 0.017174 | 0.420045 | 35.9 |
| Liaoning | 2006 | 0.559846 | 18.74 | 0.84249 | 1.976 | 0.589792 | 0.016185 | 0.568964 | 59.86 |
| Liaoning | 2007 | 0.622114 | 24.26 | 0.821055 | 2.4022 | 0.591903 | 0.01607 | 0.743523 | 90.97 |
| Liaoning | 2008 | 0.681845 | 28.25 | 0.741085 | 2.8185 | 0.600464 | 0.015662 | 1.00191 | 120.19 |
| Liaoning | 2009 | 0.670561 | 32.65 | 0.761412 | 2.9611 | 0.603548 | 0.018126 | 1.22925 | 154.44 |
| Liaoning | 2010 | 0.663418 | 46.45 | 0.730388 | 3.1888 | 0.621029 | 0.020689 | 1.6043 | 207.5 |
| Liaoning | 2011 | 0.720093 | 68.12 | 0.729227 | 3.7353 | 0.640557 | 0.022244 | 1.77263 | 242.67 |
| Liaoning | 2012 | 0.758662 | 109.3 | 0.798074 | 4.0778 | 0.656457 | 0.021901 | 2.18363 | 267.931 |
| Liaoning | 2013 | 0.884349 | 142.05 | 0.872558 | 4.3956 | 0.664605 | 0.023213 | 2.51077 | 290.4 |
| Liaoning | 2014 | 0.918468 | 102.14 | 0.994037 | 4.5915 | 0.670491 | 0.021732 | 2.47308 | 274.233 |
| Liaoning | 2015 | 0.946087 | 37.63 | 1.175838 | 4.6482 | 0.680498 | 0.017981 | 1.79179 | 51.8516 |
| Liaoning | 2016 | 0.978198 | 29.84 | 1.358506 | 4.7069 | 0.688699 | 0.018276 | 0.669225 | 29.9902 |
| Liaoning | 2017 | 1.015352 | 42.28 | 1.376148 | 5.0221 | 0.694805 | 0.019817 | 0.667674 | 53.3508 |
| Liaoning | 2018 | 1.053291 | 41.32 | 1.374848 | 5.4657 | 0.702633 | 0.01957 | 0.692378 | 48.9571 |
| Liaoning | 2019 | 1.046583 | 42.45 | 1.393155 | 5.8019 | 0.712181 | 0.022088 | 0.694455 | 33.2292 |
| Liaoning | 2020 | 1.048718 | 41.02 | 1.428704 | 5.8629 | 0.721504 | 0.02195 | 0.712511 | 25.1511 |
| Liaoning | 2021 | 1.10584 | 51.51 | 1.306975 | 6.4992 | 0.728068 | 0.021501 | 0.731036 | 32 |
| Jilin | 2005 | 0.52235 | 1.81 | 1.152733 | 1.0237 | 0.525037 | 0.014155 | 0.174109 | 6.61 |
| Jilin | 2006 | 0.546117 | 2.39 | 1.357182 | 1.1864 | 0.529563 | 0.012676 | 0.259434 | 7.61 |
| Jilin | 2007 | 0.622252 | 3.48 | 1.250254 | 1.4966 | 0.531502 | 0.012475 | 0.365136 | 8.85 |
| Jilin | 2008 | 0.6695 | 4.15 | 1.221029 | 1.7696 | 0.532187 | 0.010921 | 0.503892 | 9.93 |
| Jilin | 2009 | 0.706023 | 4.5 | 1.212155 | 1.9858 | 0.533212 | 0.014959 | 0.64116 | 11.4 |
| Jilin | 2010 | 0.7726 | 4.8 | 1.205367 | 2.337 | 0.533309 | 0.011824 | 0.78704 | 12.8 |
| Jilin | 2011 | 0.849672 | 6.06 | 1.261309 | 2.827 | 0.533945 | 0.01152 | 0.744171 | 14.81 |
| Jilin | 2012 | 0.918984 | 15.32 | 1.256998 | 3.2005 | 0.545219 | 0.012653 | 0.951154 | 16.4865 |
| Jilin | 2013 | 1.090511 | 14.78 | 1.289298 | 3.5139 | 0.557346 | 0.012696 | 0.997926 | 18.1949 |
| Jilin | 2014 | 1.164339 | 13.39 | 1.285579 | 3.7539 | 0.56813 | 0.013114 | 1.13396 | 19.66 |
| Jilin | 2015 | 1.427066 | 10 | 1.279275 | 3.8128 | 0.576349 | 0.014115 | 1.27053 | 21.27 |
| Jilin | 2016 | 1.514232 | 7.31 | 1.382965 | 4.0259 | 0.587456 | 0.013398 | 1.39232 | 22.74 |
| Jilin | 2017 | 1.587269 | 8.48 | 1.459442 | 4.289 | 0.596991 | 0.011719 | 1.32839 | 21.8 |

| Jilin | 2018 | 1.607686 | 10.82 | 1.491201 | 4.4925 | 0.608696 | 0.010219 | 1.34964 | 23.544 |
| --- | --- | --- | --- | --- | --- | --- | --- | --- | --- |
| Jilin | 2019 | 1.644251 | 10.45 | 1.52479 | 4.7554 | 0.616422 | 0.013601 | 1.131 | 19.03 |
| Jilin | 2020 | 1.70561 | 8.68 | 1.477604 | 5.0561 | 0.626511 | 0.013014 | 1.22487 | 28.9752 |
| Jilin | 2021 | 1.806321 | 11.93 | 1.43483 | 5.5148 | 0.633684 | 0.013955 | 1.35961 | 30.5911 |
| Heilongjiang | 2005 | 0.590857 | 11.14 | 0.536591 | 1.2456 | 0.53089 | 0.010281 | 0.173727 | 15.22 |
| Heilongjiang | 2006 | 0.610448 | 14.52 | 0.533656 | 1.3947 | 0.53492 | 0.010695 | 0.2236 | 17.49 |
| Heilongjiang | 2007 | 0.653323 | 15.37 | 0.547118 | 1.6023 | 0.538964 | 0.010773 | 0.28335 | 21.69 |
| Heilongjiang | 2008 | 0.714893 | 14.05 | 0.540127 | 1.8654 | 0.553987 | 0.012153 | 0.365597 | 26.56 |
| Heilongjiang | 2009 | 0.689702 | 15.31 | 0.656743 | 1.8871 | 0.554888 | 0.015113 | 0.502883 | 25.09 |
| Heilongjiang | 2010 | 0.7396 | 15.34 | 0.692313 | 2.1694 | 0.556744 | 0.014804 | 0.68126 | 27.59 |
| Heilongjiang | 2011 | 0.819821 | 23.73 | 0.675955 | 2.6093 | 0.564781 | 0.012964 | 0.747538 | 34.57 |
| Heilongjiang | 2012 | 0.863456 | 68.47 | 0.744441 | 2.9352 | 0.568743 | 0.013254 | 0.969475 | 39.914 |
| Heilongjiang | 2013 | 0.999642 | 75.27 | 0.789379 | 3.2068 | 0.580469 | 0.013908 | 1.14531 | 46.4231 |
| Heilongjiang | 2014 | 1.018059 | 108.33 | 0.945612 | 3.3464 | 0.592295 | 0.013253 | 0.982899 | 51.5551 |
| Heilongjiang | 2015 | 1.052774 | 53.75 | 1.286231 | 3.2759 | 0.604704 | 0.01349 | 1.01829 | 55.4509 |
| Heilongjiang | 2016 | 1.074526 | 40.74 | 1.478169 | 3.4025 | 0.611031 | 0.012821 | 1.06483 | 58.9647 |
| Heilongjiang | 2017 | 1.093711 | 53.59 | 1.655974 | 3.5887 | 0.619006 | 0.011906 | 1.1292 | 58.5717 |
| Heilongjiang | 2018 | 1.123339 | 66.55 | 1.784304 | 3.8199 | 0.634506 | 0.010509 | 1.07613 | 59.4792 |
| Heilongjiang | 2019 | 1.166213 | 64.91 | 1.846405 | 4.1156 | 0.646083 | 0.012788 | 1.14392 | 5.4324 |
| Heilongjiang | 2020 | 1.182931 | 48 | 1.953245 | 4.2432 | 0.655945 | 0.012704 | 1.1851 | 5.4435 |
| Heilongjiang | 2021 | 1.218724 | 65.06 | 1.788401 | 4.7199 | 0.65696 | 0.013096 | 1.26095 | 6 |
| Shanghai | 2005 | 1.118182 | 0 | 1.11108 | 4.9377 | 0.891005 | 0.022659 | 0.350966 | 68.5 |
| Shanghai | 2006 | 1.194148 | 0 | 1.131664 | 5.4996 | 0.886965 | 0.024418 | 0.390004 | 71.07 |
| Shanghai | 2007 | 1.331758 | 0 | 1.250868 | 6.3951 | 0.886628 | 0.023877 | 0.442037 | 79.2 |
| Shanghai | 2008 | 1.424159 | 0 | 1.321229 | 6.9154 | 0.886035 | 0.024448 | 0.482315 | 100.84 |
| Shanghai | 2009 | 1.518455 | 0 | 1.52718 | 7.2363 | 0.885973 | 0.026889 | 0.504375 | 105.38 |
| Shanghai | 2010 | 1.599428 | 0 | 1.394249 | 7.9396 | 0.892749 | 0.026887 | 0.51089 | 111.21 |
| Shanghai | 2011 | 1.775408 | 0 | 1.433893 | 8.5897 | 0.893039 | 0.029871 | 0.496207 | 126.01 |
| Shanghai | 2012 | 1.875138 | 0 | 1.590646 | 8.9613 | 0.892872 | 0.030485 | 0.511762 | 151.85 |
| Shanghai | 2013 | 2.045191 | 0 | 1.784348 | 9.5746 | 0.895833 | 0.033477 | 0.564779 | 167.8 |
| Shanghai | 2014 | 2.279716 | 0 | 1.911726 | 10.2827 | 0.892987 | 0.034112 | 0.601643 | 181.66 |
| Shanghai | 2015 | 2.459702 | 0 | 2.182597 | 10.9186 | 0.885273 | 0.034816 | 0.63527 | 184.59 |
| Shanghai | 2016 | 2.658513 | 0.01 | 2.473968 | 12.1369 | 0.89015 | 0.035109 | 0.675588 | 185.14 |
| Shanghai | 2017 | 2.892725 | 0.01 | 2.444735 | 13.3489 | 0.890916 | 0.036604 | 0.72466 | 170.08 |
| Shanghai | 2018 | 3.144037 | 0.01 | 2.465669 | 14.5767 | 0.891313 | 0.037743 | 0.762342 | 173 |
| Shanghai | 2019 | 3.247914 | 0 | 2.716106 | 15.3299 | 0.892382 | 0.042532 | 0.801222 | 190.48 |
| Shanghai | 2020 | 3.510337 | 0 | 2.787622 | 15.6803 | 0.893087 | 0.041467 | 0.883748 | 202.33 |
| Shanghai | 2021 | 3.736465 | 0 | 2.831992 | 17.542 | 0.89313 | 0.041687 | 0.954447 | 225.51 |
| Jiangsu | 2005 | 1.055565 | 2.83 | 0.634848 | 2.3984 | 0.505008 | 0.014889 | 0.816538 | 131.83 |
| Jiangsu | 2006 | 1.11553 | 3.88 | 0.648931 | 2.7868 | 0.518939 | 0.016294 | 1.00692 | 174.31 |
| Jiangsu | 2007 | 1.240612 | 4.32 | 0.674286 | 3.3798 | 0.532047 | 0.016554 | 1.22681 | 218.92 |
| Jiangsu | 2008 | 1.39192 | 5.5 | 0.698266 | 3.9967 | 0.54303 | 0.018772 | 1.53006 | 251.2 |
| Jiangsu | 2009 | 1.453933 | 8.22 | 0.731813 | 4.4272 | 0.556082 | 0.020365 | 1.89499 | 253.23 |
| Jiangsu | 2010 | 1.605664 | 10.06 | 0.783441 | 5.2787 | 0.605795 | 0.02073 | 2.31843 | 284.98 |
| Jiangsu | 2011 | 1.770243 | 12.48 | 0.820381 | 6.1464 | 0.620092 | 0.021816 | 2.66926 | 321.32 |
| Jiangsu | 2012 | 1.861428 | 21.93 | 0.858531 | 6.6533 | 0.630049 | 0.023982 | 3.08542 | 357.596 |
| Jiangsu | 2013 | 2.032139 | 23.39 | 0.917772 | 7.2768 | 0.643921 | 0.025062 | 3.63733 | 332.592 |
| Jiangsu | 2014 | 2.170928 | 25.33 | 0.971835 | 7.8711 | 0.657046 | 0.025494 | 4.19386 | 281.742 |
| Jiangsu | 2015 | 2.345951 | 26.48 | 1.016778 | 8.5871 | 0.674925 | 0.025278 | 4.62469 | 242.747 |
| Jiangsu | 2016 | 2.478401 | 17.56 | 1.092122 | 9.2658 | 0.689297 | 0.026204 | 4.96632 | 245.43 |
| Jiangsu | 2017 | 2.717227 | 14 | 1.091412 | 10.2202 | 0.701769 | 0.02632 | 5.3277 | 251.354 |
| Jiangsu | 2018 | 2.946344 | 13.95 | 1.114103 | 11.0508 | 0.711935 | 0.026869 | 5.62073 | 255.925 |
| Jiangsu | 2019 | 3.033167 | 7.13 | 1.168812 | 11.665 | 0.724643 | 0.030468 | 5.90738 | 261.242 |
| Jiangsu | 2020 | 3.146614 | 6.23 | 1.201823 | 12.1333 | 0.734458 | 0.029238 | 5.92511 | 283.84 |
| Jiangsu | 2021 | 3.576965 | 9.06 | 1.138842 | 13.8255 | 0.739447 | 0.029291 | 6.26876 | 288.5 |
| Zhejiang | 2005 | 1.082834 | 3.37 | 0.746825 | 2.6277 | 0.560208 | 0.012534 | 0.652007 | 77.23 |
| Zhejiang | 2006 | 1.157642 | 4.65 | 0.734585 | 3.0415 | 0.565063 | 0.014638 | 0.759022 | 88.89 |
| Zhejiang | 2007 | 1.283382 | 6.11 | 0.745641 | 3.6454 | 0.572066 | 0.015107 | 0.842043 | 103.66 |

| Zhejiang | 2008 | 1.408934 | 6.75 | 0.755566 | 4.1061 | 0.575979 | 0.01619 | 0.9323 | 100.73 |
| --- | --- | --- | --- | --- | --- | --- | --- | --- | --- |
| Zhejiang | 2009 | 1.466812 | 6.68 | 0.826155 | 4.3543 | 0.579037 | 0.017465 | 1.07423 | 99.4 |
| Zhejiang | 2010 | 1.624632 | 6.36 | 0.844083 | 5.111 | 0.616119 | 0.018037 | 1.2376 | 110.02 |
| Zhejiang | 2011 | 1.786858 | 8.5 | 0.863413 | 5.7828 | 0.62298 | 0.018776 | 1.41853 | 116.66 |
| Zhejiang | 2012 | 1.902083 | 8.41 | 0.92316 | 6.1097 | 0.629024 | 0.021017 | 1.76494 | 130.693 |
| Zhejiang | 2013 | 2.002912 | 9.23 | 0.960926 | 6.5105 | 0.63935 | 0.021891 | 2.07821 | 141.59 |
| Zhejiang | 2014 | 2.125922 | 9.3 | 0.955849 | 6.8569 | 0.649576 | 0.022684 | 2.42628 | 157.973 |
| Zhejiang | 2015 | 2.218649 | 11.53 | 1.02539 | 7.3276 | 0.663158 | 0.023242 | 2.73233 | 169.602 |
| Zhejiang | 2016 | 2.330539 | 11.76 | 1.102966 | 7.8384 | 0.677207 | 0.023926 | 3.02761 | 175.775 |
| Zhejiang | 2017 | 2.491826 | 12.58 | 1.171026 | 8.5612 | 0.689141 | 0.024165 | 3.1696 | 179.021 |
| Zhejiang | 2018 | 2.676023 | 12.83 | 1.213793 | 9.323 | 0.700143 | 0.024925 | 3.39464 | 186.387 |
| Zhejiang | 2019 | 2.789354 | 12.1 | 1.295682 | 9.877 | 0.715765 | 0.029777 | 3.73411 | 135.592 |
| Zhejiang | 2020 | 2.62324 | 12.84 | 1.371747 | 10.0738 | 0.721707 | 0.028731 | 3.93575 | 157.848 |
| Zhejiang | 2021 | 2.781011 | 14.01 | 1.304135 | 11.3839 | 0.726606 | 0.029142 | 4.36081 | 185.766 |
| Anhui | 2005 | 0.872412 | 4.57 | 1.143169 | 0.9193 | 0.355065 | 0.008087 | 0.252511 | 6.88 |
| Anhui | 2006 | 0.919501 | 6.05 | 1.068936 | 1.063 | 0.371031 | 0.009123 | 0.353356 | 13.94 |
| Anhui | 2007 | 1.026133 | 7.43 | 1.02423 | 1.2989 | 0.387055 | 0.009041 | 0.508753 | 29.99 |
| Anhui | 2008 | 1.143209 | 9.85 | 0.956038 | 1.5535 | 0.405053 | 0.010328 | 0.674696 | 34.9 |
| Anhui | 2009 | 1.221315 | 11.56 | 0.927765 | 1.7715 | 0.420975 | 0.012518 | 0.899073 | 38.84 |
| Anhui | 2010 | 1.36503 | 12.65 | 0.821326 | 2.1923 | 0.430082 | 0.012355 | 1.15429 | 50.14 |
| Anhui | 2011 | 1.540638 | 14.52 | 0.760499 | 2.7303 | 0.447924 | 0.013178 | 1.24557 | 66.29 |
| Anhui | 2012 | 1.614878 | 17.92 | 0.76506 | 3.0697 | 0.463031 | 0.015364 | 1.54258 | 86.3811 |
| Anhui | 2013 | 1.75986 | 19.74 | 0.799089 | 3.4404 | 0.478624 | 0.017106 | 1.86219 | 106.877 |
| Anhui | 2014 | 1.87492 | 20.82 | 0.841522 | 3.758 | 0.49308 | 0.017478 | 2.18756 | 123.398 |
| Anhui | 2015 | 1.937338 | 20.6 | 0.979563 | 3.9692 | 0.509732 | 0.018119 | 2.4386 | 136.195 |
| Anhui | 2016 | 2.077525 | 17.82 | 1.067984 | 4.3686 | 0.526272 | 0.018059 | 2.70334 | 147.671 |
| Anhui | 2017 | 2.279453 | 21.45 | 1.136541 | 4.9092 | 0.542843 | 0.019035 | 2.92751 | 158.965 |
| Anhui | 2018 | 2.558172 | 22.47 | 1.225912 | 5.6063 | 0.556452 | 0.019082 | 3.27295 | 170.016 |
| Anhui | 2019 | 2.656489 | 28.21 | 1.2665 | 6.0561 | 0.570256 | 0.02397 | 3.57406 | 179.367 |
| Anhui | 2020 | 2.589588 | 30.72 | 1.292025 | 6.2411 | 0.583292 | 0.023205 | 3.75634 | 183.054 |
| Anhui | 2021 | 2.774309 | 33.46 | 1.273838 | 6.9676 | 0.59398 | 0.023637 | 4.10944 | 193 |
| Fujian | 2005 | 1.044597 | 2.14 | 0.816241 | 1.8107 | 0.493956 | 0.008355 | 0.231672 | 62.3 |
| Fujian | 2006 | 1.09384 | 3.18 | 0.829297 | 2.0915 | 0.504045 | 0.009024 | 0.298182 | 71.85 |
| Fujian | 2007 | 1.229134 | 4.31 | 0.852006 | 2.5915 | 0.51412 | 0.008814 | 0.428775 | 81.31 |
| Fujian | 2008 | 1.324419 | 5.46 | 0.825821 | 3.0153 | 0.530091 | 0.009321 | 0.520768 | 100.26 |
| Fujian | 2009 | 1.392717 | 5.87 | 0.845181 | 3.3999 | 0.551009 | 0.010903 | 0.62312 | 57.37 |
| Fujian | 2010 | 1.529538 | 6.46 | 0.782241 | 4.0773 | 0.57108 | 0.011391 | 0.81991 | 58.03 |
| Fujian | 2011 | 1.682003 | 8.09 | 0.763047 | 4.7928 | 0.581131 | 0.012362 | 0.991089 | 62.01 |
| Fujian | 2012 | 1.805088 | 9.33 | 0.763256 | 5.2959 | 0.593075 | 0.013422 | 1.24399 | 63.3774 |
| Fujian | 2013 | 2.01108 | 9.12 | 0.758392 | 5.8255 | 0.607979 | 0.013958 | 1.53274 | 66.7896 |
| Fujian | 2014 | 2.059677 | 11.96 | 0.753599 | 6.3709 | 0.620025 | 0.014233 | 1.81779 | 71.1499 |
| Fujian | 2015 | 2.260769 | 11.45 | 0.811819 | 6.7649 | 0.632279 | 0.01465 | 2.13014 | 76.8339 |
| Fujian | 2016 | 2.46007 | 9.25 | 0.870394 | 7.4024 | 0.643924 | 0.015343 | 2.32373 | 81.9465 |
| Fujian | 2017 | 2.695532 | 9.89 | 0.941516 | 8.3758 | 0.657811 | 0.016048 | 2.64163 | 85.7672 |
| Fujian | 2018 | 2.946295 | 12.22 | 0.926421 | 9.4719 | 0.669834 | 0.016615 | 2.94542 | 44.5777 |
| Fujian | 2019 | 3.085479 | 9.17 | 0.98007 | 10.2722 | 0.678753 | 0.019902 | 3.11919 | 46.0953 |
| Fujian | 2020 | 3.136138 | 7.26 | 1.026819 | 10.5106 | 0.687575 | 0.019317 | 3.10672 | 50.2347 |
| Fujian | 2021 | 3.270069 | 9.86 | 1.001136 | 11.875 | 0.696919 | 0.018994 | 3.29312 | 53.4007 |
| Jiangxi | 2005 | 0.91955 | 2.29 | 0.757018 | 0.9172 | 0.369984 | 0.007231 | 0.21766 | 24.23 |
| Jiangxi | 2006 | 1.007876 | 3.46 | 0.677661 | 1.0859 | 0.386725 | 0.008048 | 0.268357 | 28.07 |
| Jiangxi | 2007 | 1.143503 | 5.37 | 0.663899 | 1.327 | 0.398123 | 0.008446 | 0.330194 | 31.04 |
| Jiangxi | 2008 | 1.288175 | 8.9 | 0.682306 | 1.5816 | 0.413636 | 0.0091 | 0.474543 | 36.04 |
| Jiangxi | 2009 | 1.312679 | 10.87 | 0.691581 | 1.7277 | 0.431859 | 0.009948 | 0.664314 | 40.24 |
| Jiangxi | 2010 | 1.476534 | 12.91 | 0.620192 | 2.1099 | 0.44061 | 0.009293 | 0.87723 | 51.01 |
| Jiangxi | 2011 | 1.672086 | 18.66 | 0.619438 | 2.5928 | 0.457532 | 0.008356 | 0.90876 | 60.59 |
| Jiangxi | 2012 | 1.770751 | 29.72 | 0.64921 | 2.8624 | 0.473966 | 0.008877 | 1.07742 | 68.2431 |
| Jiangxi | 2013 | 1.885838 | 34.7 | 0.665331 | 3.1952 | 0.490393 | 0.009475 | 1.28503 | 75.5096 |
| Jiangxi | 2014 | 1.945015 | 46.75 | 0.704274 | 3.4988 | 0.50558 | 0.009772 | 1.50793 | 84.5074 |

| Jiangxi | 2015 | 1.992271 | 47.97 | 0.800555 | 3.7436 | 0.523077 | 0.010321 | 1.73881 | 94.7321 |
| --- | --- | --- | --- | --- | --- | --- | --- | --- | --- |
| Jiangxi | 2016 | 2.106369 | 56.62 | 0.900315 | 4.095 | 0.539813 | 0.011273 | 1.96942 | 104.406 |
| Jiangxi | 2017 | 2.252653 | 59.64 | 0.945609 | 4.4878 | 0.557083 | 0.012657 | 2.20853 | 114.637 |
| Jiangxi | 2018 | 2.446317 | 44.88 | 1.067135 | 5.0347 | 0.573454 | 0.013677 | 2.45368 | 125.717 |
| Jiangxi | 2019 | 2.55223 | 30.5 | 1.089554 | 5.464 | 0.590788 | 0.01746 | 2.67942 | 135.8 |
| Jiangxi | 2020 | 2.62851 | 27.41 | 1.119041 | 5.7065 | 0.604337 | 0.016705 | 2.89913 | 146.022 |
| Jiangxi | 2021 | 2.883167 | 24.42 | 1.078023 | 6.602 | 0.614567 | 0.016836 | 3.21224 | 157.8 |
| Shandong | 2005 | 0.660025 | 18.24 | 0.585696 | 1.7308 | 0.450043 | 0.012234 | 0.93073 | 89.71 |
| Shandong | 2006 | 0.708831 | 26.14 | 0.596215 | 2.0443 | 0.460952 | 0.012342 | 1.11114 | 100.01 |
| Shandong | 2007 | 0.778641 | 28.99 | 0.617556 | 2.4329 | 0.467492 | 0.013747 | 1.25377 | 110.12 |
| Shandong | 2008 | 0.886694 | 28.81 | 0.62492 | 2.8861 | 0.476054 | 0.016 | 1.54359 | 82.02 |
| Shandong | 2009 | 0.911184 | 32.81 | 0.662381 | 3.1282 | 0.48321 | 0.017589 | 1.90345 | 80.1 |
| Shandong | 2010 | 0.974567 | 33.29 | 0.720579 | 3.5599 | 0.496975 | 0.01981 | 2.32805 | 91.68 |
| Shandong | 2011 | 1.052055 | 38.36 | 0.771365 | 4.0581 | 0.508639 | 0.021615 | 2.67497 | 111.6 |
| Shandong | 2012 | 1.104322 | 91.11 | 0.828844 | 4.4348 | 0.520293 | 0.023751 | 3.1256 | 123.527 |
| Shandong | 2013 | 1.339014 | 92.62 | 0.896462 | 4.8673 | 0.534578 | 0.024835 | 3.67891 | 140.531 |
| Shandong | 2014 | 1.390672 | 119.57 | 0.954892 | 5.1933 | 0.547716 | 0.025684 | 4.24955 | 151.951 |
| Shandong | 2015 | 1.405695 | 103.81 | 1.030474 | 5.6205 | 0.569734 | 0.025814 | 4.83124 | 163.009 |
| Shandong | 2016 | 1.464012 | 95.18 | 1.109611 | 5.9239 | 0.591297 | 0.026651 | 5.33229 | 168.256 |
| Shandong | 2017 | 1.571452 | 99.56 | 1.160747 | 6.2993 | 0.607894 | 0.02782 | 5.52027 | 178.573 |
| Shandong | 2018 | 1.642367 | 119.75 | 1.241646 | 6.6284 | 0.614568 | 0.024656 | 5.7466 | 205.164 |
| Shandong | 2019 | 1.704289 | 119.98 | 1.322305 | 6.9901 | 0.618642 | 0.023843 | 5.27538 | 146.893 |
| Shandong | 2020 | 1.740468 | 108.27 | 1.369702 | 7.1825 | 0.630497 | 0.023104 | 5.4653 | 176.476 |
| Shandong | 2021 | 1.857726 | 126.15 | 1.34041 | 8.151 | 0.63943 | 0.023465 | 5.79321 | 215.2 |
| Henan | 2005 | 0.70043 | 8.71 | 0.614574 | 1.0978 | 0.306503 | 0.005428 | 0.431163 | 12.3 |
| Henan | 2006 | 0.7379 | 16.59 | 0.600345 | 1.2761 | 0.324744 | 0.006662 | 0.590471 | 18.45 |
| Henan | 2007 | 0.831073 | 21.31 | 0.602707 | 1.5811 | 0.343376 | 0.00682 | 0.801011 | 30.62 |
| Henan | 2008 | 0.934636 | 24.1 | 0.560741 | 1.8879 | 0.360272 | 0.006896 | 1.04906 | 40.33 |
| Henan | 2009 | 0.971129 | 24.23 | 0.599616 | 2.028 | 0.377042 | 0.009113 | 1.37045 | 47.99 |
| Henan | 2010 | 1.05678 | 26.08 | 0.604132 | 2.3984 | 0.385008 | 0.009322 | 1.65859 | 62.47 |
| Henan | 2011 | 1.141221 | 26.59 | 0.638144 | 2.7901 | 0.404714 | 0.01005 | 1.7769 | 100.82 |
| Henan | 2012 | 1.224754 | 34.13 | 0.687527 | 3.0497 | 0.419849 | 0.010731 | 2.145 | 121.178 |
| Henan | 2013 | 1.443807 | 37.4 | 0.738331 | 3.3114 | 0.436018 | 0.011232 | 2.60875 | 134.566 |
| Henan | 2014 | 1.510481 | 39.19 | 0.784552 | 3.5982 | 0.450492 | 0.011569 | 3.07822 | 149.269 |
| Henan | 2015 | 1.659764 | 35.35 | 0.842477 | 3.8338 | 0.470158 | 0.01173 | 3.56603 | 160.864 |
| Henan | 2016 | 1.803042 | 28.07 | 0.905825 | 4.1326 | 0.48783 | 0.012278 | 4.04151 | 169.931 |
| Henan | 2017 | 2.022602 | 35.57 | 0.942933 | 4.5723 | 0.505647 | 0.012986 | 4.44969 | 172.243 |
| Henan | 2018 | 2.2038 | 60.29 | 1.070222 | 5.0714 | 0.522405 | 0.013447 | 4.81012 | 179.021 |
| Henan | 2019 | 2.40887 | 68.88 | 1.130706 | 5.4356 | 0.540147 | 0.016778 | 5.19493 | 187.273 |
| Henan | 2020 | 2.384819 | 67.06 | 1.200874 | 5.4691 | 0.55427 | 0.016611 | 5.41831 | 200.648 |
| Henan | 2021 | 2.470971 | 77.96 | 1.225397 | 5.8587 | 0.564505 | 0.017545 | 5.66213 | 210.73 |
| Hubei | 2005 | 0.641711 | 3.29 | 0.9573 | 1.1342 | 0.432049 | 0.011592 | 0.267658 | 21.85 |
| Hubei | 2006 | 0.681668 | 4.25 | 0.958634 | 1.321 | 0.438082 | 0.012534 | 0.334347 | 24.49 |
| Hubei | 2007 | 0.778334 | 4.56 | 0.966625 | 1.6593 | 0.44306 | 0.011776 | 0.433036 | 27.66 |
| Hubei | 2008 | 0.895115 | 5.36 | 0.930717 | 2.0153 | 0.451935 | 0.012959 | 0.564701 | 32.45 |
| Hubei | 2009 | 0.962379 | 6.89 | 0.901218 | 2.3081 | 0.459965 | 0.016176 | 0.786689 | 36.58 |
| Hubei | 2010 | 1.071961 | 8.4 | 0.830569 | 2.8359 | 0.497032 | 0.016275 | 1.02627 | 40.5 |
| Hubei | 2011 | 1.202861 | 10.13 | 0.789169 | 3.4719 | 0.517882 | 0.016197 | 1.25573 | 46.55 |
| Hubei | 2012 | 1.278152 | 12.43 | 0.785781 | 3.9149 | 0.532261 | 0.01702 | 1.55783 | 56.6591 |
| Hubei | 2013 | 1.616111 | 16.23 | 0.898846 | 4.3835 | 0.545016 | 0.017582 | 1.93073 | 68.8847 |
| Hubei | 2014 | 1.730493 | 18.83 | 0.940398 | 4.8635 | 0.557256 | 0.01809 | 2.29153 | 79.2792 |
| Hubei | 2015 | 1.960587 | 17.69 | 1.007008 | 5.2021 | 0.571795 | 0.018511 | 2.65639 | 89.4801 |
| Hubei | 2016 | 2.098069 | 15.56 | 1.061437 | 5.6844 | 0.585726 | 0.017989 | 3.00117 | 101.289 |
| Hubei | 2017 | 2.301298 | 14.15 | 1.144986 | 6.3169 | 0.598747 | 0.018816 | 3.22824 | 109.939 |
| Hubei | 2018 | 2.519003 | 16.01 | 1.189258 | 7.1097 | 0.609937 | 0.019564 | 3.58334 | 119.41 |
| Hubei | 2019 | 2.623527 | 20.11 | 1.222908 | 7.6712 | 0.618357 | 0.022129 | 3.96676 | 129.075 |
| Hubei | 2020 | 2.646268 | 15.46 | 1.439556 | 7.3687 | 0.628895 | 0.023377 | 3.22101 | 103.519 |
| Hubei | 2021 | 2.753474 | 21.73 | 1.351322 | 8.6551 | 0.640823 | 0.023162 | 3.87809 | 76.1733 |

| Hunan | 2005 | 0.656064 | 1.22 | 1.12497 | 1.02 | 0.37006 | 0.006986 | 0.262907 | 20.72 |
| --- | --- | --- | --- | --- | --- | --- | --- | --- | --- |
| Hunan | 2006 | 0.70236 | 2.06 | 1.04141 | 1.1733 | 0.387102 | 0.007212 | 0.317552 | 25.93 |
| Hunan | 2007 | 0.798479 | 2.83 | 0.996587 | 1.4626 | 0.404563 | 0.007926 | 0.415476 | 32.71 |
| Hunan | 2008 | 0.915185 | 3.37 | 0.960082 | 1.7758 | 0.421473 | 0.009967 | 0.553404 | 40.05 |
| Hunan | 2009 | 0.958125 | 3.85 | 0.997743 | 1.9979 | 0.431939 | 0.012018 | 0.770338 | 45.98 |
| Hunan | 2010 | 1.046655 | 6.14 | 0.919215 | 2.4005 | 0.433029 | 0.011981 | 0.96636 | 51.84 |
| Hunan | 2011 | 1.170421 | 6.79 | 0.856792 | 2.8766 | 0.449628 | 0.012329 | 1.18809 | 61.5 |
| Hunan | 2012 | 1.266549 | 8.45 | 0.877704 | 3.2203 | 0.462215 | 0.013566 | 1.45232 | 72.8034 |
| Hunan | 2013 | 1.578254 | 9.7 | 0.920147 | 3.5702 | 0.476364 | 0.013888 | 1.78414 | 87.0482 |
| Hunan | 2014 | 1.689729 | 10.47 | 0.962799 | 3.9181 | 0.48979 | 0.014215 | 2.12429 | 102.659 |
| Hunan | 2015 | 1.966281 | 10.23 | 1.036263 | 4.3155 | 0.507937 | 0.014461 | 2.50451 | 115.644 |
| Hunan | 2016 | 2.078377 | 8.86 | 1.1587 | 4.6606 | 0.526943 | 0.015194 | 2.83533 | 128.521 |
| Hunan | 2017 | 2.225533 | 9.59 | 1.290502 | 5.103 | 0.546208 | 0.016806 | 3.19592 | 144.749 |
| Hunan | 2018 | 2.337217 | 11.31 | 1.391057 | 5.4763 | 0.560965 | 0.01812 | 3.51552 | 161.913 |
| Hunan | 2019 | 2.493225 | 10.65 | 1.353435 | 6.0104 | 0.574548 | 0.022527 | 3.87058 | 181.013 |
| Hunan | 2020 | 2.552541 | 11.43 | 1.338794 | 6.2537 | 0.58766 | 0.021633 | 4.16475 | 209.978 |
| Hunan | 2021 | 2.703341 | 15.44 | 1.318465 | 6.8913 | 0.597101 | 0.022508 | 4.49793 | 228.548 |
| Guangdong | 2005 | 1.225547 | 2.43 | 0.861474 | 2.3997 | 0.606809 | 0.0111 | 0.697793 | 123.64 |
| Guangdong | 2006 | 1.299951 | 4.18 | 0.859439 | 2.7861 | 0.630057 | 0.012056 | 0.797337 | 145.11 |
| Guangdong | 2007 | 1.428746 | 5.94 | 0.877298 | 3.3236 | 0.631366 | 0.012737 | 0.929426 | 171.26 |
| Guangdong | 2008 | 1.563466 | 6.86 | 0.878214 | 3.7543 | 0.63368 | 0.013693 | 1.08687 | 191.67 |
| Guangdong | 2009 | 1.600762 | 7.84 | 0.930004 | 3.9418 | 0.634057 | 0.016546 | 1.29331 | 195.35 |
| Guangdong | 2010 | 1.707469 | 9.35 | 0.908812 | 4.4669 | 0.661814 | 0.017602 | 1.56237 | 202.61 |
| Guangdong | 2011 | 1.863512 | 11.12 | 0.931096 | 5.0076 | 0.665675 | 0.019699 | 1.70692 | 217.98 |
| Guangdong | 2012 | 1.956069 | 12.05 | 0.985526 | 5.2308 | 0.671497 | 0.021685 | 1.87515 | 235.491 |
| Guangdong | 2013 | 2.194665 | 13.96 | 1.032069 | 5.6029 | 0.680923 | 0.023095 | 2.23084 | 249.521 |
| Guangdong | 2014 | 2.303666 | 15.45 | 1.039887 | 5.9909 | 0.686222 | 0.023549 | 2.62939 | 268.714 |
| Guangdong | 2015 | 2.481403 | 16.55 | 1.109545 | 6.4516 | 0.695068 | 0.024062 | 3.0343 | 268.755 |
| Guangdong | 2016 | 2.632508 | 16.7 | 1.215901 | 6.9671 | 0.701461 | 0.024769 | 3.33036 | 233.492 |
| Guangdong | 2017 | 2.836631 | 14.18 | 1.284511 | 7.6218 | 0.707438 | 0.025572 | 3.77617 | 229.067 |
| Guangdong | 2018 | 2.998656 | 13.46 | 1.321555 | 8.1625 | 0.718092 | 0.027062 | 4.18023 | 223.087 |
| Guangdong | 2019 | 3.162876 | 11.12 | 1.389684 | 8.6956 | 0.726479 | 0.032225 | 4.64423 | 218.674 |
| Guangdong | 2020 | 3.221513 | 11.72 | 1.425884 | 8.8521 | 0.741524 | 0.031308 | 4.97862 | 215.399 |
| Guangdong | 2021 | 3.387145 | 17.94 | 1.368369 | 9.8561 | 0.746295 | 0.031336 | 5.29227 | 212.957 |
| Guangxi | 2005 | 0.768624 | 1.97 | 1.142566 | 0.8069 | 0.336266 | 0.003902 | 0.166117 | 3.79 |
| Guangxi | 2006 | 0.819576 | 2.53 | 1.114726 | 0.9421 | 0.346472 | 0.00412 | 0.219872 | 4.47 |
| Guangxi | 2007 | 0.912865 | 3.17 | 1.053954 | 1.1542 | 0.362416 | 0.004018 | 0.293967 | 6.84 |
| Guangxi | 2008 | 0.993589 | 4.14 | 1.041038 | 1.3471 | 0.381645 | 0.005081 | 0.375641 | 9.71 |
| Guangxi | 2009 | 1.005362 | 5.34 | 1.094575 | 1.4708 | 0.392092 | 0.006636 | 0.523724 | 10.35 |
| Guangxi | 2010 | 1.079989 | 6.97 | 0.994921 | 1.807 | 0.4 | 0.007355 | 0.70576 | 9.12 |
| Guangxi | 2011 | 1.198867 | 8.56 | 0.955461 | 2.2234 | 0.418904 | 0.007864 | 0.799066 | 10.14 |
| Guangxi | 2012 | 1.234758 | 10.19 | 1.037974 | 2.4181 | 0.43481 | 0.008599 | 0.980861 | 7.4853 |
| Guangxi | 2013 | 1.3679 | 11.97 | 1.156945 | 2.6416 | 0.451067 | 0.008652 | 1.19077 | 7.0008 |
| Guangxi | 2014 | 1.427989 | 17.1 | 1.171642 | 2.8603 | 0.465409 | 0.008235 | 1.38432 | 10.0119 |
| Guangxi | 2015 | 1.509056 | 18.06 | 1.269041 | 3.089 | 0.479942 | 0.007156 | 1.62278 | 17.2208 |
| Guangxi | 2016 | 1.594125 | 17.22 | 1.369027 | 3.334 | 0.492485 | 0.007303 | 1.82368 | 8.8845 |
| Guangxi | 2017 | 1.701482 | 16.78 | 1.429402 | 3.6441 | 0.505808 | 0.007993 | 2.04991 | 8.2272 |
| Guangxi | 2018 | 1.813527 | 16.69 | 1.481256 | 3.9837 | 0.518294 | 0.007382 | 2.2713 | 5.059 |
| Guangxi | 2019 | 1.884392 | 16.64 | 1.53284 | 4.2778 | 0.529707 | 0.008156 | 2.48935 | 11.0946 |
| Guangxi | 2020 | 1.873692 | 18.09 | 1.621743 | 4.4237 | 0.541941 | 0.00783 | 2.5939 | 15.6931 |
| Guangxi | 2021 | 1.91137 | 22.59 | 1.485089 | 5.0137 | 0.550725 | 0.007912 | 2.79104 | 16.4412 |
| Hainan | 2005 | 1.076262 | 0.57 | 1.589208 | 1.0753 | 0.451691 | 0.001808 | 0.036717 | 6.84 |
| Hainan | 2006 | 1.116299 | 0.86 | 1.457819 | 1.235 | 0.460526 | 0.002044 | 0.042389 | 7.49 |
| Hainan | 2007 | 1.167456 | 0.77 | 1.491392 | 1.4683 | 0.472189 | 0.002107 | 0.050237 | 11.2 |
| Hainan | 2008 | 1.298917 | 1.28 | 1.547504 | 1.7357 | 0.480094 | 0.002238 | 0.070542 | 12.83 |
| Hainan | 2009 | 1.314623 | 1.03 | 1.710916 | 1.886 | 0.491898 | 0.00358 | 0.098832 | 9.38 |
| Hainan | 2010 | 1.487295 | 1.24 | 1.835225 | 2.3323 | 0.498274 | 0.003464 | 0.1317 | 15.12 |
| Hainan | 2011 | 1.539278 | 1.58 | 1.721883 | 2.8014 | 0.503371 | 0.004221 | 0.165723 | 15.23 |

| Hainan | 2012 | 1.652507 | 2.85 | 1.817853 | 3.0993 | 0.50989 | 0.004911 | 0.214538 | 16.4119 |
| --- | --- | --- | --- | --- | --- | --- | --- | --- | --- |
| Hainan | 2013 | 1.811221 | 2.66 | 2.18591 | 3.4053 | 0.522826 | 0.00475 | 0.269793 | 18.106 |
| Hainan | 2014 | 1.895131 | 2.63 | 2.213309 | 3.7166 | 0.53312 | 0.0049 | 0.311223 | 19.1558 |
| Hainan | 2015 | 1.948956 | 2.68 | 2.283384 | 3.9704 | 0.549206 | 0.004553 | 0.345122 | 24.6567 |
| Hainan | 2016 | 2.061593 | 3.16 | 2.501659 | 4.3009 | 0.567398 | 0.005305 | 0.389045 | 22.1561 |
| Hainan | 2017 | 2.16226 | 3.41 | 2.547571 | 4.6631 | 0.580247 | 0.005136 | 0.42444 | 23.0598 |
| Hainan | 2018 | 2.262995 | 3.68 | 2.726807 | 5.0263 | 0.59165 | 0.005478 | 0.371385 | 8.1876 |
| Hainan | 2019 | 2.354594 | 3.73 | 2.923141 | 5.3929 | 0.59397 | 0.006866 | 0.337218 | 15.2021 |
| Hainan | 2020 | 2.451379 | 3.58 | 3.131878 | 5.5438 | 0.602767 | 0.006575 | 0.364195 | 30.3324 |
| Hainan | 2021 | 2.65887 | 3.66 | 3.230819 | 6.3991 | 0.609804 | 0.007225 | 0.401343 | 24.6236 |
| Chongqing | 2005 | 0.697648 | 2.99 | 0.919767 | 1.2335 | 0.452109 | 0.00928 | 0.193316 | 5.16 |
| Chongqing | 2006 | 0.726516 | 3.77 | 0.879257 | 1.3915 | 0.46688 | 0.009461 | 0.240736 | 6.96 |
| Chongqing | 2007 | 0.802217 | 4.27 | 0.922541 | 1.6966 | 0.482955 | 0.009852 | 0.312774 | 10.29 |
| Chongqing | 2008 | 0.911491 | 4.96 | 1.015424 | 2.0865 | 0.499824 | 0.010204 | 0.397959 | 24.52 |
| Chongqing | 2009 | 0.946141 | 5.16 | 1.012132 | 2.3346 | 0.515915 | 0.011953 | 0.521428 | 33.76 |
| Chongqing | 2010 | 1.026705 | 5.02 | 1.046246 | 2.8084 | 0.529983 | 0.012436 | 0.66889 | 30.43 |
| Chongqing | 2011 | 1.155738 | 8.01 | 1.049111 | 3.4864 | 0.549932 | 0.012636 | 0.747338 | 58.26 |
| Chongqing | 2012 | 1.249719 | 8.69 | 1.018745 | 3.918 | 0.566387 | 0.013781 | 0.873617 | 35.2418 |
| Chongqing | 2013 | 1.618475 | 8.4 | 1.018218 | 4.3527 | 0.582863 | 0.013548 | 1.04352 | 41.4353 |
| Chongqing | 2014 | 1.70188 | 9.71 | 1.012385 | 4.8311 | 0.597437 | 0.013806 | 1.22854 | 42.3348 |
| Chongqing | 2015 | 2.070543 | 11.76 | 1.077248 | 5.248 | 0.614658 | 0.015399 | 1.43532 | 37.7183 |
| Chongqing | 2016 | 2.257955 | 11.34 | 1.161653 | 5.8327 | 0.633441 | 0.016767 | 1.60481 | 27.9037 |
| Chongqing | 2017 | 2.423759 | 14.78 | 1.222377 | 6.4171 | 0.650127 | 0.01817 | 1.7537 | 22.6042 |
| Chongqing | 2018 | 2.52294 | 13.7 | 1.285642 | 6.846 | 0.66614 | 0.019001 | 1.87646 | 32.503 |
| Chongqing | 2019 | 2.655619 | 14.42 | 1.34819 | 7.4337 | 0.682246 | 0.022317 | 1.98155 | 23.6529 |
| Chongqing | 2020 | 2.821712 | 13.79 | 1.330876 | 7.8294 | 0.694609 | 0.021037 | 2.05883 | 21.0119 |
| Chongqing | 2021 | 3.028391 | 13.25 | 1.331702 | 8.745 | 0.7033 | 0.021049 | 2.18442 | 22.36 |
| Sichuan | 2005 | 0.608989 | 3.9 | 0.9562 | 0.8828 | 0.330005 | 0.013424 | 0.358518 | 8.87 |
| Sichuan | 2006 | 0.654118 | 5.31 | 0.880949 | 1.0371 | 0.343004 | 0.01269 | 0.441288 | 12.08 |
| Sichuan | 2007 | 0.743067 | 7.83 | 0.865464 | 1.2963 | 0.355974 | 0.01317 | 0.56398 | 14.93 |
| Sichuan | 2008 | 0.842268 | 8.72 | 0.841204 | 1.5685 | 0.374048 | 0.012566 | 0.712781 | 30.88 |
| Sichuan | 2009 | 0.869429 | 10 | 0.808183 | 1.7387 | 0.387049 | 0.015116 | 1.13719 | 35.9 |
| Sichuan | 2010 | 0.962719 | 12.83 | 0.791566 | 2.123 | 0.40174 | 0.015344 | 1.31167 | 60.25 |
| Sichuan | 2011 | 1.06878 | 21.84 | 0.817014 | 2.6136 | 0.418527 | 0.013971 | 1.42222 | 94.81 |
| Sichuan | 2012 | 1.162693 | 24.95 | 0.850211 | 2.9627 | 0.433519 | 0.014668 | 1.704 | 98.01 |
| Sichuan | 2013 | 1.380283 | 26.97 | 0.873 | 3.275 | 0.449624 | 0.015084 | 2.03261 | 102.844 |
| Sichuan | 2014 | 1.453383 | 29.1 | 0.938942 | 3.5563 | 0.465045 | 0.015551 | 2.33186 | 102.876 |
| Sichuan | 2015 | 1.657489 | 28.72 | 1.022445 | 3.715 | 0.482674 | 0.016574 | 2.55259 | 99.9607 |
| Sichuan | 2016 | 1.766821 | 26.18 | 1.173805 | 4.0297 | 0.500061 | 0.016941 | 2.8812 | 79.7687 |
| Sichuan | 2017 | 1.971247 | 30.41 | 1.309166 | 4.5835 | 0.517795 | 0.016826 | 3.19021 | 81.0135 |
| Sichuan | 2018 | 2.154152 | 52.52 | 1.396141 | 5.1658 | 0.535032 | 0.017181 | 3.51561 | 89.6375 |
| Sichuan | 2019 | 2.229994 | 63.31 | 1.417759 | 5.5619 | 0.553586 | 0.022761 | 3.81795 | 30.7178 |
| Sichuan | 2020 | 2.289334 | 73.59 | 1.453204 | 5.8009 | 0.567316 | 0.021758 | 3.92486 | 24.5237 |
| Sichuan | 2021 | 2.505466 | 83.06 | 1.4274 | 6.461 | 0.578237 | 0.023641 | 4.15642 | 33.6 |
| Guizhou | 2005 | 0.343877 | 1.98 | 0.977065 | 0.5218 | 0.268633 | 0.00567 | 0.099825 | 1.96 |
| Guizhou | 2006 | 0.366807 | 3.07 | 0.990556 | 0.6103 | 0.274526 | 0.006404 | 0.119743 | 1.84 |
| Guizhou | 2007 | 0.418767 | 4.32 | 1.035799 | 0.7778 | 0.282489 | 0.004811 | 0.14888 | 1.53 |
| Guizhou | 2008 | 0.494707 | 5.33 | 1.074077 | 0.9697 | 0.291157 | 0.005393 | 0.186445 | 1.74 |
| Guizhou | 2009 | 0.509719 | 8.49 | 1.111563 | 1.0814 | 0.298841 | 0.006845 | 0.241202 | 1.34 |
| Guizhou | 2010 | 0.552754 | 11.01 | 1.150931 | 1.2882 | 0.338028 | 0.006639 | 0.31049 | 2.95 |
| Guizhou | 2011 | 0.619287 | 11.95 | 1.176281 | 1.6024 | 0.350425 | 0.006464 | 0.423592 | 5.15 |
| Guizhou | 2012 | 0.682521 | 12.72 | 1.164164 | 1.8947 | 0.362977 | 0.006185 | 0.57178 | 4.9116 |
| Guizhou | 2013 | 0.857457 | 15.33 | 1.199212 | 2.2089 | 0.378855 | 0.00592 | 0.73736 | 5.7673 |
| Guizhou | 2014 | 0.944825 | 17.05 | 1.202909 | 2.5101 | 0.402502 | 0.00605 | 0.902575 | 4.6565 |
| Guizhou | 2015 | 1.128104 | 24.67 | 1.209998 | 2.8547 | 0.429612 | 0.00591 | 1.09455 | 4.1941 |
| Guizhou | 2016 | 1.227608 | 23.15 | 1.222235 | 3.1589 | 0.455561 | 0.006224 | 1.3204 | 9.6739 |
| Guizhou | 2017 | 1.38182 | 30.77 | 1.32819 | 3.5988 | 0.477518 | 0.007049 | 1.55039 | 8.2439 |
| Guizhou | 2018 | 1.529813 | 32.6 | 1.396789 | 4.0271 | 0.49529 | 0.00792 | 1.79535 | 6.0845 |

| Guizhou | 2019 | 1.608875 | 34 | 1.426325 | 4.3727 | 0.514813 | 0.009643 | 1.81151 | 6.7887 |
| --- | --- | --- | --- | --- | --- | --- | --- | --- | --- |
| Guizhou | 2020 | 1.681542 | 29.57 | 1.446192 | 4.6355 | 0.531623 | 0.009054 | 1.86947 | 4.3907 |
| Guizhou | 2021 | 1.727531 | 39.21 | 1.441822 | 5.0476 | 0.543354 | 0.009268 | 1.81152 | 4.4385 |
| Yunnan | 2005 | 0.58063 | 3.19 | 1.055359 | 0.789 | 0.295056 | 0.00609 | 0.177763 | 1.74 |
| Yunnan | 2006 | 0.617878 | 4.61 | 1.022578 | 0.9158 | 0.30493 | 0.005109 | 0.22086 | 3.02 |
| Yunnan | 2007 | 0.711855 | 6.3 | 1.054924 | 1.1287 | 0.315906 | 0.005101 | 0.275903 | 3.95 |
| Yunnan | 2008 | 0.801058 | 8.31 | 1.025241 | 1.3286 | 0.329958 | 0.005152 | 0.343593 | 7.77 |
| Yunnan | 2009 | 0.81852 | 10.49 | 1.120481 | 1.4427 | 0.339969 | 0.005658 | 0.452637 | 9.1 |
| Yunnan | 2010 | 0.891763 | 12.92 | 1.03354 | 1.6866 | 0.347023 | 0.005714 | 0.55287 | 13.29 |
| Yunnan | 2011 | 0.998199 | 14.08 | 1.132156 | 2.0653 | 0.365801 | 0.005891 | 0.6191 | 17.38 |
| Yunnan | 2012 | 1.063613 | 17.7 | 1.121165 | 2.3992 | 0.384798 | 0.0062 | 0.783113 | 21.89 |
| Yunnan | 2013 | 1.273371 | 18.75 | 1.189181 | 2.7665 | 0.399914 | 0.006222 | 0.99683 | 25.15 |
| Yunnan | 2014 | 1.343082 | 17.13 | 1.238296 | 3.0217 | 0.412207 | 0.006117 | 1.14985 | 27.06 |
| Yunnan | 2015 | 1.435012 | 18.23 | 1.345485 | 3.2117 | 0.429337 | 0.007313 | 1.35006 | 29.92 |
| Yunnan | 2016 | 1.526105 | 17.24 | 1.460852 | 3.5051 | 0.44644 | 0.008113 | 1.61194 | 8.67 |
| Yunnan | 2017 | 1.655858 | 24.56 | 1.55589 | 3.9458 | 0.462817 | 0.008536 | 1.8936 | 9.63 |
| Yunnan | 2018 | 1.801605 | 29.43 | 1.529343 | 4.4446 | 0.474378 | 0.00897 | 2.11326 | 10.56 |
| Yunnan | 2019 | 1.910166 | 29.62 | 1.504355 | 4.9323 | 0.486636 | 0.010593 | 2.29288 | 7.2 |
| Yunnan | 2020 | 1.891528 | 31.54 | 1.497049 | 5.2047 | 0.500424 | 0.010018 | 2.46944 | 7.59 |
| Yunnan | 2021 | 2.023007 | 41.11 | 1.446242 | 5.7717 | 0.510448 | 0.010125 | 2.56821 | 7.8 |
| Shaanxi | 2005 | 0.685149 | 6.82 | 0.879965 | 1.0357 | 0.372358 | 0.024206 | 0.188218 | 6.28 |
| Shaanxi | 2006 | 0.749768 | 11.45 | 0.818318 | 1.2439 | 0.391187 | 0.022065 | 0.248069 | 9.25 |
| Shaanxi | 2007 | 0.838659 | 14.53 | 0.786908 | 1.5342 | 0.406149 | 0.021419 | 0.341502 | 11.95 |
| Shaanxi | 2008 | 0.96769 | 16.95 | 0.762172 | 1.9331 | 0.420925 | 0.019964 | 0.461442 | 13.7 |
| Shaanxi | 2009 | 0.994306 | 17.9 | 0.803659 | 2.1485 | 0.434934 | 0.023757 | 0.62469 | 15.11 |
| Shaanxi | 2010 | 1.10843 | 22.3 | 0.754609 | 2.6388 | 0.457564 | 0.022092 | 0.79637 | 18.2 |
| Shaanxi | 2011 | 1.247351 | 49.44 | 0.694508 | 3.2467 | 0.473572 | 0.024156 | 0.943108 | 23.55 |
| Shaanxi | 2012 | 1.330961 | 61.59 | 0.685115 | 3.7453 | 0.497227 | 0.020308 | 1.20446 | 29.3609 |
| Shaanxi | 2013 | 1.499027 | 78.77 | 0.715598 | 4.1906 | 0.515773 | 0.021546 | 1.48841 | 36.78 |
| Shaanxi | 2014 | 1.550685 | 84.9 | 0.750757 | 4.561 | 0.53018 | 0.021077 | 1.71919 | 41.7557 |
| Shaanxi | 2015 | 1.523821 | 90.7 | 0.881114 | 4.6654 | 0.547322 | 0.021968 | 1.85822 | 46.2118 |
| Shaanxi | 2016 | 1.568072 | 82.67 | 0.947982 | 4.9341 | 0.564017 | 0.022031 | 2.08253 | 50.1178 |
| Shaanxi | 2017 | 1.711172 | 144.66 | 0.950979 | 5.5216 | 0.580686 | 0.021464 | 2.38194 | 58.9437 |
| Shaanxi | 2018 | 1.855961 | 181.31 | 0.971566 | 6.1115 | 0.59654 | 0.022237 | 2.62966 | 68.4794 |
| Shaanxi | 2019 | 1.913726 | 230.42 | 1.020638 | 6.5506 | 0.61283 | 0.024514 | 2.6954 | 77.2947 |
| Shaanxi | 2020 | 1.925222 | 235.37 | 1.116058 | 6.5867 | 0.626549 | 0.024306 | 2.80591 | 84.4315 |
| Shaanxi | 2021 | 2.075179 | 373.06 | 0.976739 | 7.6171 | 0.636318 | 0.022705 | 2.72174 | 98.3594 |
| Gansu | 2005 | 0.42691 | 2.11 | 1.020591 | 0.7332 | 0.300196 | 0.010512 | 0.087036 | 0.2 |
| Gansu | 2006 | 0.464469 | 3.12 | 0.926067 | 0.8653 | 0.310954 | 0.010894 | 0.102259 | 0.3 |
| Gansu | 2007 | 0.523575 | 4.2 | 0.84132 | 1.0501 | 0.322606 | 0.009607 | 0.130416 | 1.18 |
| Gansu | 2008 | 0.574544 | 5.55 | 0.874537 | 1.2048 | 0.335555 | 0.010353 | 0.171278 | 1.28 |
| Gansu | 2009 | 0.596231 | 5.13 | 0.934428 | 1.2802 | 0.348728 | 0.011413 | 0.2363 | 1.34 |
| Gansu | 2010 | 0.665814 | 6.18 | 0.817002 | 1.5421 | 0.361328 | 0.010625 | 0.31583 | 1.35 |
| Gansu | 2011 | 0.741543 | 14.36 | 0.875076 | 1.8846 | 0.372649 | 0.010069 | 0.396579 | 0.7 |
| Gansu | 2012 | 0.769669 | 16.62 | 0.926042 | 2.1141 | 0.387843 | 0.011218 | 0.514503 | 0.611 |
| Gansu | 2013 | 0.825405 | 19.49 | 1.003141 | 2.3647 | 0.404809 | 0.011123 | 0.652794 | 0.7129 |
| Gansu | 2014 | 0.866642 | 22.98 | 1.062374 | 2.5724 | 0.422758 | 0.011797 | 0.788413 | 1.0032 |
| Gansu | 2015 | 0.875497 | 16.81 | 1.32426 | 2.5946 | 0.442331 | 0.012613 | 0.875423 | 1.1036 |
| Gansu | 2016 | 0.946288 | 13.02 | 1.45911 | 2.7396 | 0.460714 | 0.012594 | 0.966399 | 1.1588 |
| Gansu | 2017 | 0.977705 | 16.73 | 1.574529 | 2.9103 | 0.481364 | 0.012049 | 0.582775 | 0.4356 |
| Gansu | 2018 | 1.035933 | 22.82 | 1.599218 | 3.2178 | 0.497018 | 0.011982 | 0.560047 | 0.5041 |
| Gansu | 2019 | 1.115157 | 23.78 | 1.675727 | 3.4707 | 0.506975 | 0.012571 | 0.59701 | 0.8205 |
| Gansu | 2020 | 1.107961 | 21.7 | 1.758248 | 3.5848 | 0.522191 | 0.012205 | 0.643577 | 0.8875 |
| Gansu | 2021 | 1.212381 | 29.46 | 1.567426 | 4.0976 | 0.533333 | 0.011696 | 0.715014 | 0.9488 |
| Qinghai | 2005 | 0.298978 | 1.42 | 1.294272 | 0.9233 | 0.392265 | 0.006007 | 0.032981 | 2.66 |
| Qinghai | 2006 | 0.307452 | 1.94 | 1.276633 | 1.0728 | 0.392336 | 0.005639 | 0.040854 | 2.75 |
| Qinghai | 2007 | 0.34372 | 2.71 | 1.319288 | 1.31 | 0.400362 | 0.005277 | 0.048284 | 3.1 |
| Qinghai | 2008 | 0.393524 | 3.94 | 1.26463 | 1.622 | 0.407942 | 0.004348 | 0.058324 | 2.2 |

| Qinghai | 2009 | 0.400183 | 6.82 | 1.281652 | 1.6907 | 0.420108 | 0.001703 | 0.079823 | 2.15 |
| --- | --- | --- | --- | --- | --- | --- | --- | --- | --- |
| Qinghai | 2010 | 0.445515 | 9.58 | 1.277353 | 2.0418 | 0.447602 | 0.008652 | 0.10169 | 2.19 |
| Qinghai | 2011 | 0.429723 | 14.91 | 1.201591 | 2.4233 | 0.464789 | 0.009194 | 0.143558 | 1.69 |
| Qinghai | 2012 | 0.433733 | 17.78 | 1.18365 | 2.6839 | 0.478109 | 0.00857 | 0.188342 | 2.0578 |
| Qinghai | 2013 | 0.454678 | 18.37 | 1.215272 | 3.0005 | 0.492119 | 0.008055 | 0.236109 | 0.93723 |
| Qinghai | 2014 | 0.462885 | 23.15 | 1.282876 | 3.2218 | 0.508681 | 0.007739 | 0.286123 | 0.501 |
| Qinghai | 2015 | 0.487515 | 21.92 | 1.367757 | 3.4883 | 0.516464 | 0.005768 | 0.321063 | 0.55 |
| Qinghai | 2016 | 0.550646 | 14.84 | 1.347586 | 3.8968 | 0.536082 | 0.0062 | 0.352805 | 0.1495 |
| Qinghai | 2017 | 0.587908 | 18.57 | 1.282156 | 4.2211 | 0.554608 | 0.007261 | 0.388355 | 0.1833 |
| Qinghai | 2018 | 0.629698 | 20.28 | 1.267441 | 4.6854 | 0.572402 | 0.006295 | 0.416705 | 0.0446 |
| Qinghai | 2019 | 0.694475 | 18.31 | 1.2872 | 4.9976 | 0.588136 | 0.007242 | 0.43754 | 0.677214 |
| Qinghai | 2020 | 0.72519 | 24.75 | 1.337124 | 5.0845 | 0.600337 | 0.007077 | 0.38416 | 0.2552 |
| Qinghai | 2021 | 0.721082 | 22.63 | 1.221294 | 5.7036 | 0.609428 | 0.006517 | 0.37302 | 0.0321 |
| Ningxia | 2005 | 0.22866 | 0.23 | 0.963433 | 0.9796 | 0.422819 | 0.005518 | 0.044325 | 1.41 |
| Ningxia | 2006 | 0.241467 | 0.61 | 0.884126 | 1.1389 | 0.430464 | 0.007317 | 0.049875 | 1.38 |
| Ningxia | 2007 | 0.285188 | 1.26 | 0.859479 | 1.4458 | 0.440984 | 0.008546 | 0.05998 | 1.7 |
| Ningxia | 2008 | 0.352775 | 1.16 | 0.838918 | 1.8554 | 0.449838 | 0.006584 | 0.082885 | 1.21 |
| Ningxia | 2009 | 0.373917 | 1.51 | 0.932884 | 2.0382 | 0.4608 | 0.00821 | 0.107591 | 1.42 |
| Ningxia | 2010 | 0.426965 | 1.92 | 0.936597 | 2.4984 | 0.478673 | 0.007317 | 0.14442 | 0.81 |
| Ningxia | 2011 | 0.447556 | 2.74 | 0.90998 | 3.0161 | 0.501543 | 0.00792 | 0.164474 | 2.02 |
| Ningxia | 2012 | 0.46708 | 4.1 | 0.956675 | 3.2609 | 0.511381 | 0.008541 | 0.209686 | 2.182 |
| Ningxia | 2013 | 0.486915 | 5 | 0.998112 | 3.5135 | 0.528529 | 0.008979 | 0.265114 | 1.4814 |
| Ningxia | 2014 | 0.500172 | 5.43 | 1.026668 | 3.6815 | 0.548673 | 0.009661 | 0.317379 | 0.9244 |
| Ningxia | 2015 | 0.474329 | 13.31 | 1.0976 | 3.7876 | 0.570175 | 0.009886 | 0.350545 | 1.8639 |
| Ningxia | 2016 | 0.497478 | 10.49 | 1.153429 | 4.0339 | 0.58705 | 0.01075 | 0.379425 | 2.5363 |
| Ningxia | 2017 | 0.495326 | 18.57 | 1.097937 | 4.5718 | 0.609929 | 0.012155 | 0.372838 | 3.114 |
| Ningxia | 2018 | 0.494394 | 25 | 1.171091 | 4.9614 | 0.621127 | 0.012991 | 0.304981 | 2.1443 |
| Ningxia | 2019 | 0.490128 | 18.9 | 1.185433 | 5.2537 | 0.635983 | 0.0159 | 0.273568 | 2.5123 |
| Ningxia | 2020 | 0.498714 | 20.14 | 1.22077 | 5.5021 | 0.649098 | 0.015065 | 0.284511 | 2.7242 |
| Ningxia | 2021 | 0.570175 | 25.53 | 1.012005 | 6.3461 | 0.66069 | 0.015352 | 0.29077 | 2.93 |
| Xinjiang | 2005 | 0.457732 | 6.08 | 0.80543 | 1.2687 | 0.371642 | 0.002539 | 0.133906 | 0.47 |
| Xinjiang | 2006 | 0.48903 | 7.95 | 0.741311 | 1.4567 | 0.379512 | 0.002874 | 0.156705 | 1.04 |
| Xinjiang | 2007 | 0.532245 | 9.47 | 0.829902 | 1.6887 | 0.391408 | 0.002857 | 0.185084 | 1.25 |
| Xinjiang | 2008 | 0.585977 | 10.89 | 0.754995 | 1.9605 | 0.396527 | 0.003862 | 0.225997 | 1.9 |
| Xinjiang | 2009 | 0.563015 | 12.28 | 0.91338 | 1.9755 | 0.398333 | 0.005145 | 0.272545 | 2.16 |
| Xinjiang | 2010 | 0.646571 | 32.47 | 0.758166 | 2.47 | 0.430206 | 0.004981 | 0.34232 | 2.37 |
| Xinjiang | 2011 | 0.658036 | 65.01 | 0.773065 | 2.9624 | 0.437303 | 0.005052 | 0.463214 | 3.35 |
| Xinjiang | 2012 | 0.626452 | 69.35 | 0.866502 | 3.3103 | 0.442077 | 0.005356 | 0.615878 | 4.0795 |
| Xinjiang | 2013 | 0.615664 | 71.74 | 1.030547 | 3.6988 | 0.449453 | 0.005421 | 0.77323 | 4.8102 |
| Xinjiang | 2014 | 0.620692 | 78.17 | 1.05202 | 4.0193 | 0.467957 | 0.005311 | 0.944774 | 4.17 |
| Xinjiang | 2015 | 0.594083 | 66.36 | 1.291663 | 3.952 | 0.487631 | 0.005587 | 1.0813 | 4.525 |
| Xinjiang | 2016 | 0.590774 | 52.62 | 1.367227 | 4.002 | 0.504119 | 0.005877 | 1.02875 | 4.0076 |
| Xinjiang | 2017 | 0.64189 | 69.06 | 1.345613 | 4.5476 | 0.518952 | 0.005108 | 1.20891 | 1.9613 |
| Xinjiang | 2018 | 0.72394 | 87.97 | 1.387121 | 5.1238 | 0.540079 | 0.00502 | 0.904266 | 2.0526 |
| Xinjiang | 2019 | 0.735376 | 90.26 | 1.469547 | 5.3542 | 0.555295 | 0.004972 | 0.926873 | 3.31 |
| Xinjiang | 2020 | 0.727049 | 82.19 | 1.469238 | 5.3606 | 0.565251 | 0.004898 | 1.07703 | 2.1634 |
| Xinjiang | 2021 | 0.837063 | 96.35 | 1.216125 | 6.2991 | 0.572422 | 0.004176 | 1.23858 | 2.37 |
